# Chronic exercise and neuropsychological function in healthy young adults: a randomised controlled trial investigating a running intervention

**DOI:** 10.1007/s10339-024-01177-1

**Published:** 2024-02-29

**Authors:** Mhairi Alexander, Liana Machado

**Affiliations:** 1 Department of Psychology, University of Otago, William James Building, 275 Leith Walk, Dunedin, 9054 New Zealand; 2 Brain Research New Zealand, Auckland, New Zealand

**Keywords:** Physical activity, Cognitive performance, Affect, Environment, Enjoyment

## Abstract

Despite the well-known physical and mental health benefits of regular exercise, many of the world’s population, including healthy young adults, grossly undershoot recommended physical activity levels. Chronic exercise has potential to improve cognitive performance and affect in most age groups. However, there is currently a poverty of relevant research in young adults, especially randomised controlled trials. To address this, the current research investigated the effects of a running intervention on neuropsychological function (cognition and affect) in young adults. We predicted that following a running intervention, neuropsychological performance would improve alongside increases in aerobic fitness. Thirty-two healthy young adult university students were randomised (using a 3:1 ratio) into an intervention or control group, with the intervention group (*n* = 24) asked to run for 30 min three times a week over a 6-week period and the control group (*n* = 8) asked to maintain their current level of exercise over a 6-week period. We assessed fitness, cognitive performance, affect and running enjoyment at baseline and follow-up, and runners recorded the environmental conditions of their runs. Repeated measures ANCOVAs failed to find any significant effects of the running intervention on fitness or the neuropsychological measures. Anecdotal evidence supported running environment and enjoyment as potentially relevant factors. The failure to find any fitness improvements, which likely underpins the lack of neuropsychological improvements, highlights the importance of monitoring exercise sessions. Coupled with other insights gained from this trial, this article may prove useful towards future endeavours to develop exercise interventions beneficial to young adults.

TRN: ACTRN12621000242820, Date of registration: 08/03/2021.

**Supplementary Information:**

The online version contains supplementary material available at 10.1007/s10339-024-01177-1.

## Introduction

Physical activity (PA) has widely researched beneficial effects on physical health, well-being and brain function (Colcombe et al. 2006; Erickson et al. 2019; Leckie et al. 2014; Stenling et al. 2021, 2022; Stillman et al. 2020; Stroth et al. 2009, 2010; Viña et al. 2012). Conversely, sedentary behaviours, especially when combined with low PA levels, have been linked to the development of serious health issues (Biswas et al. 2015; Ekelund et al. 2019; Patterson et al. 2018). Focusing on neuropsychological function—defined here as cognition and affect—chronic exercise (i.e., regular engagement over a prolonged period of time, as opposed to a single bout, termed acute exercise; Sellami et al. 2018) has been found to confer benefits in most age groups, evidenced by the exercise leading to improved cognitive performance and more positive and less negative affect (Erickson et al. 2019; Guiney and Machado 2013; Stillman et al. 2020; Stroth et al. 2010; Williams and Tappen 2007). Despite this, in 2016, more than 25% of the global population were not meeting recommended PA levels (Guthold et al. 2018). Of particular relevance here, young adult university students have been found to spend more time sedentary than the general population (Castro et al. 2020). Given that PA habits established earlier in life tend to continue into later life (Hirvensalo and Lintunen 2011), it is important to assist younger people to establish good PA habits. However, few chronic exercise studies investigating links with cognitive function and affect have looked at young adults, leaving a need for more randomised controlled trial research in this understudied age group.

Neuropsychological functioning is a fundamental part of everyday life, as it supports academic achievement and most daily activities, including social interactions. Affect refers to the valence (positive or negative) of how one if feeling. Cognitive flexibility, inhibitory control and transient memory are key components of cognition and are part of a set of higher order cognitive functions known as executive functions (Diamond 2013; Machado 2021). Inhibitory control allows an individual to disengage from irrelevant stimuli and supports cognitive flexibility, which facilitates the ability to engage with relevant information, enabling efficient transitions between tasks, goals and mental states whilst also facilitating adaptation and creative thinking (Diamond 2013; Ionescu 2011; Karbach and Unger 2014; Machado et al. 2013). Regarding transient memory, storage and processing components are highly interrelated (Cowan 2008) but have different developmental progressions and utilise different neural areas, marking them as distinct cognitive domains (Diamond 2013; Park et al. 2002; Postle et al. 2006). Individuals can store limited quantities of information in an easily accessible fashion over a short period of time (Cowan 2008). Processing components enable individuals to manipulate the stored information, thus supporting executive functioning (Alloway and Alloway 2010; Diamond 2013). Both domains have separable systems, although they coordinate functionally, related to verbal versus spatial information (Baddeley 2003; Park et al. 2002; Ramos et al. 2020; but see Shevlin 2020), evidenced by distinct patterns of activation during verbal versus visuospatial tasks as well as different developmental progressions and vulnerabilities to brain deterioration and disease (Donolato et al. 2017; Kouwenhoven and Machado 2023; Ramos et al. 2022; Ramos and Machado 2021). In relation to this, the current research separated out visuospatial and verbal content as well as storage and processing demands.

### Chronic exercise and neuropsychological function

Although a number of studies have observed improvements in neuropsychological functions (cognition and affect) across a range of exercise modalities in healthy and clinical samples of adults (Awick et al. 2017; Dilorenzo et al. 1999; Hansen et al. 2001; Kramer and Colcombe 2003; Oken et al. 2006; Pontifex et al. 2013; Williams and Tappen 2007), studies in young adults have seen less consistent results. Conflicts in the literature indicate that a minimum regularity of exercise may be required to observe improvements in young adults. For example, with regard to affect, Gallego et al. (2015) found no significant improvements in depression or anxiety scores following an 8-week PA regime with a single 1-h session per week, whereas Wipfli et al. (2011) found significant improvements in depression scores and decreases in serotonin levels after a 7-week cycling regime that included three 1-h sessions per week. However, the dearth of chronic exercise research in young adults focusing on affect and cognition limits conclusions, to the extent that authors of a recent review reported insufficient data with respect to determining relationships between chronic exercise and cognitive function in young adults and called for more research in this age group (Erickson et al. 2019). Considered alongside evidence that young adults tend to have less positive affect profiles (Machado et al. 2019), more young adult research is warranted.

Many of the studies that have been conducted in young adults used somewhat limited methodological practices, such as relying solely on self-report measures of PA, that can lead to inaccurate results (Padilla et al. 2014, 2013; Weinstein et al. 2012). Fitness tests offer a solution to the uncertainty of self-report data and enable objective assessment of whether increased fitness, which results from regular exercise, is connected with cognition and affect, although fitness tests cannot separate out other factors such as genetics that can influence fitness (Rankinen et al. 2001). Submaximal fitness tests vary in their accuracy but can provide valid measures of maximal fitness (Castro-Piñero et al. 2021; Chung and Lee 2022) and do not require participants to exercise to maximum, instead using equations to estimate a participant’s VO_2MAX_ (Lee et al. 2019; Reed et al. 2020). Another issue with studies-to-date conducted in young adults relates the use of cross-sectional designs involving data collection at only one time point and in most cases making comparisons between subsets of the participants, for example categorised based on their reported physical activity levels (Hillman et al. 2006; Kamijo and Takeda 2010). However, this does not allow causal relationships to be established between chronic exercise and neuropsychological function, which requires an exercise intervention. The following paragraphs summarise two studies that reported positive neuropsychological outcomes in young adults following a chronic exercise intervention (Stroth et al. 2009, 2010) and that provided the foundation for the current study.

In the first study, 28 young adults (17–29 years) were asked to either run three times a week for 30 min at a time or maintain their usual exercise regime over 6 weeks (Stroth et al. 2009). Runners were given intensity levels to run at based on lactate threshold. After two exclusions, results showed in the runners statistically significant benefits with large effect sizes in positive affect scores and visuospatial short-term memory, but no statistically significant changes in concentration, verbal short-term memory or negative affect. The second study used a similar methodology, but included a wider age range (17–47 years), investigated different cognitive skills and allowed people to choose whether to join the running or control group (Stroth et al. 2010). In this study, 75 participants completed a 4-month intervention period with runners completing up to 50 sessions and asked to exercise no more than once a day and up to four times a week. Runners were given a tailored exercise regime that entailed exercising until they reached a prescribed intensity level based on lactate threshold. Results indicated statistically significant improvements in positive affect and in two executive functions (cognitive control and cognitive flexibility), but not in verbal working memory or negative affect.

While the Stroth studies (2009, 2010) indicate that there are relationships between chronic exercise and both cognitive function and affect in young adults, there were several limitations that must be addressed. In their first study, Stroth et al. (2009) examined short-term memory using the Visual and Verbal Memory test, which has been validated but is not commonly known or widely used and does not assess manipulation of stored information (executive functioning). More commonplace tests of visuospatial and verbal short-term memory storage and manipulation could inform on whether benefits generalise beyond the specific test used by these investigators and may shed further light on the nature of short-term memory benefits. In their second study (Stroth et al. 2010), allowing participants to choose which exercise condition (control or running) they participated in, rather than being randomised, reflects a more real-life situation, but the lack of randomisation compromises determination of whether the intervention caused the changes observed.

In sum, while relationships have been established between chronic exercise and neuropsychological function in young adults, more randomised controlled trial research is required to confirm and further characterise the links.

### Environment and enjoyment

Relationships between environment and well-being indicate that time spent in natural environments can benefit life satisfaction, positive affect and cognition, specifically EF, which suggests that exercising in natural environments may confer additional neuropsychological benefits (Berman et al. 2008; White et al. 2019a, b). The Attention Restoration Theory suggests that natural environments can be cognitively restorative by presenting a way of attending that requires less effort (Kaplan and Berman 2010). Thus, performance of tasks which use directed attention (e.g., those tapping working memory or cognitive flexibility) and are cognitively tiring should improve the most (Kaplan and Berman 2010). However, Trammell and Aguilar (2020) observed that while executive functioning improved following exercise, no interaction was found between exercise and environment nor were there any benefits to affect, which contradicts other studies that indicate exercise in a natural environment should additionally benefit cognition and affect (Berman et al. 2008; Leppämäki et al. 2002; White et al. 2019a, b). However, as the former study utilised an acute exercise intervention and the latter studies did not directly relate environment to exercise-related change in neuropsychological measures, the influence of environmental conditions of a chronic exercise intervention on neuropsychological function requires further exploration.

Regarding enjoyment, research in young adults has established a positive relationship between the degree of enjoyment of an acute exercise intervention and the extent to which positive affect increased (Raedeke 2007). Furthermore, it has subsequently been posited that greater enjoyment of an exercise intervention will promote adherence (Bartlett et al. 2011; Greene et al. 2018), which should enhance exercise-induced neuropsychological benefits. Thus, it is important to consider participants’ enjoyment of the exercise intervention when assessing neuropsychological benefits.

### Current research

The current research aimed to advance our understanding of the effects of regular, long-term exercise on cognition and affect in sedentary (i.e., not exercising more than twice a week) young adults, whilst also addressing some of the methodological issues in the existing literature. Our secondary focus was to assess the influences of enjoyment of the exercise intervention and the exercise environment on neuropsychological outcomes. Based on Stroth et al. (2009), the intervention consisted of a 6-week running regime where the runner group was asked to run three times a week for 30 min at a time and the control group was asked to continue their existing exercise regime. The intention to enrol sedentary participants stemmed from a combination of the ethical advantages of increasing rather than decreasing participant PA as well as the expectation that sedentary young adults would stand to benefit most with regard to cardiorespiratory fitness (Budde et al. 2012; Cui et al. 2020; Tsai et al. 2016), thus serving to promote a healthier lifestyle in those more in need. We assessed affect via the Positive and Negative Affect Scale (PANAS), as per Stroth et al. (2009, 2010), and cognitive performance using a computerised battery that included tests (assessing basic visuomotor function, inhibition, and mental flexibility) previously shown to be sensitive to PA in healthy young adults (Cameron et al. 2015; Guiney et al. 2015; Shoemaker et al. 2020, 2019; Stenling et al. 2019) and also commonly used tests of spatial and verbal short-term memory storage capacity (Forward Spatial and Forward Digit) and manipulation (Backward Spatial and Backward Digit). The chosen cognitive tests have previously been demonstrated to show sensitivity to both brain disease and individual differences in healthy adults (Barbey et al. 2013; Brett and Machado 2017; Flannery et al. 2018; Kouwenhoven and Machado 2023; Ramos et al. 2022; Shoemaker et al. 2021). Sex was included as a covariate based on previous research showing that it can influence effects of exercise on cognitive performance (Lennox et al. 2019; Ludyga et al. 2020; Stenling et al. 2019).

Based on previous findings (Stroth et al. 2009, 2010), we anticipated that improvements would be observed in cognitive performance and positive affect scores of the runner group compared to the control group, along with increased cardiorespiratory fitness (Padilla et al. 2014; Silverman and Deuster 2014). We also predicted that enjoyment of the exercise regime would have a positive relationship with improvements in positive affect scores as well as with other neuropsychological improvements in relation to increased adherence (Bartlett et al. 2011; Greene et al. 2018; Raedeke 2007), and that exercising in outdoor environments would be more beneficial to cognitive performance and affect scores, as has been indicated by previous research (Berman et al. 2008; Leppämäki et al. 2002; White et al. 2019a, b).

## Method

The University of Otago Human Ethics Committee approved the study (reference code 20/128) and data collection took place between 10/03/2021 and 20/08/2021. All participants read an information sheet before giving informed written consent, ahead of taking part.

### Participants

In association with a psychology course, 88 University of Otago students were assessed for eligibility. Inclusion criteria: age 18–30 years, currently sedentary (defined as not exercising more than twice a week), normal or corrected to normal vision, not colour blind, physically capable of running or jogging. Potential participants were screened by email, containing the inclusion criteria, the date of their follow-up session (which they were asked to ensure they could attend) and the Physical Activity Readiness Questionnaire (PAR-Q) (Warburton et al. 2011) to ensure that it was safe to commence physical activity (note: participants completed a second PAR-Q during the first testing session and we sent a reminder email repeating key information the day before). Participants were asked not to consume alcohol or caffeine for at least 12 h before testing sessions.

**Fig. 1:**
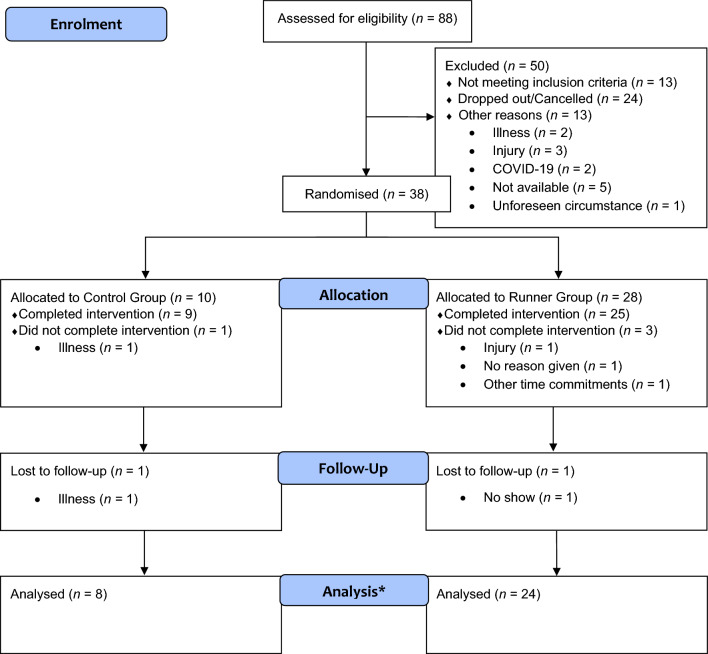
Consort flowchart depicting the recruitment, allocation and exclusions for the study. Note. * One runner was removed from the Backwards Digit analysis and 12 participants (runner *n* = 11, control *n* = 1) were removed for a subsample analysis of fitness. Please refer to the results section for further explanation

Fifty individuals were excluded from the study or dropped out prior to commencing the first session, a further four did not complete the intervention and two did not attend their follow-up session (see Fig. 1 for details). Thus, the total number of completed participants was 32 (*M*age = 20.31 years, *SD* = 2.61, range = 18–28; 25 females; 30 right-handed, *M*education = 14.02 years, *SD* = 1.73, range = 11–21). Figure 1 depicts a Consolidated Standard of Reporting Trials (CONSORT) flow chart summarizing recruitment, allocation and exclusions.

### Design

This was a pre-registered randomised controlled parallel intervention trial (Identifier No. ACTRN12621000242820: refer to CONSORT checklist in Online Resource 1). The independent variable involved a 6-week exercise intervention and the dependent measures related to cognitive performance, affect, fitness, and within the runner group only, enjoyment and environmental conditions of the running. Each participant attended two sessions (one prior to the 6-week intervention and one just after) in a laboratory setting and at the end of the first session was randomly allocated into either the runner or control group using a 3:1 ratio. The 3:1 ratio was used in an effort to ensure sufficient runners for the analyses specific to the runner group.

### Procedure overview

Upon arrival to the first session, the participant’s general information was collected including date of birth, years of education, handedness. At this point, participants all confirmed that they met all of the inclusion criteria, did not have any neurological or psychological disorders and that they had not consumed any caffeine, medications or alcohol in the last 12 h. Participants were randomised into one of the groups using Randola (http://www.rando.la/), an online site that allows for randomisation with minimisation using a ratio allocation (Riegle van West et al. 2019). Participants completed the PANAS, an ethnicity question and the New Zealand Physical Activity Questionnaire—Short Form (NZPAQ-SF), after which height and weight were measured for a body mass index (BMI) calculation (weight/height^2^). Participants then completed a cognitive battery, followed by a 3-min Young Men’s Christian Association (YMCA) step test. Participants wore a Polar M430 Heart Rate Watch during the fitness test and were shown how to begin a new time lap on the watch, which was used to indicate the different phases of the fitness test: start, end, and 1 min heart rate count period start and end.

Finally, participants were taken through the procedure for the next 6 weeks, after which the follow-up date was confirmed. Participants assigned to the control group were asked to maintain their current exercise regime, whereas those assigned to the runner group were asked to run three times a week for 30 min. Runners were advised that if they were not able to run continuously (especially initially) they could briskly walk or jog at a pace that increased their heart and breathing rate. Whilst it was important they aim to complete the full regime, runners were told that their health and safety was paramount and they could miss a running session if they were ill or injured. A record of exercise was sent to all participants at the beginning of each study week (e.g., if they started the study on Tuesday, they would receive the next record on the following Monday in advance of the new study week), which they completed and returned at the end of the week via email. The follow-up session followed the same procedure as the first testing session. Time of day was controlled across sessions, such that a given participant completed both sessions during the same time period, morning (9 am to 12 pm) or afternoon (12 to 5 pm).

### Materials and apparatus

#### PANAS

Affect was assessed using the PANAS (Watson et al. 1988), which asks participants to self-report the extent to which they had felt a particular way (e.g., distressed, proud, strong, irritable) over the week prior to the session. It includes 20 feeling terms, 10 negative (e.g., afraid) and 10 positive (e.g., inspired), which participants rate on a five-point scale: *very slightly or not at all (1), a little (2), moderately (3), quite a bit (4)* and *extremely (5)*.

#### New Zealand Physical Activity Questionnaire

Participants’ PA levels over the past 7 days were assessed using NZPAQ—SF (McLean and Tobias 2004), a validated measure of weekly PA (Moy et al. 2008). This self-report questionnaire assesses duration (minutes and hours per week), intensity (brisk walking, moderate and vigorous) and frequency (days per week) of PA that lasted more than 10 min over the previous week. To assess whether the participant was not chronically active, the participant was asked to reflect on whether they had been regularly physically active over the last 6 months, which was defined as engaging in a minimum of 15 min of vigorous activity or 30 min of moderate activity five or more days a week, including brisk walking.

#### Cognitive battery

This was completed on a Dell Optiplex 7050 desktop PC (Ubuntu 16.04 operating system, 600 × 340 mm monitor) using MATLAB 2016b (The MathWorks, Natick, MA) and Psychophysics toolbox 3 (Brainard 1997; Pelli 1997). All participants were seated with their head in a chin rest 57 cm away from the screen in a dark room. The cognitive battery took approximately 20 min to complete and included the following tasks in this order: Pro, Anti, Pro/Anti, Forward Spatial, Backward Spatial, Forward Digit and Backward Digit. In the second session, for runners the battery included at the end a visual analogue scale that enquired about enjoyment of the running. Participants responded using a button box (DirectIN Rotary Controller with Buttons, Empirisoft Corporation; buttons 2 × 2 cm and spaced 2 cm apart) for the Pro, Anti and Pro/Anti tasks; using a mouse for the running enjoyment visual analogue scale and Forward and Backward Spatial tasks; and verbally for the Forward and Backward Digit tasks. Before each of the tasks, participants were given both verbal and written instructions, as well as the opportunity to ask any questions.

##### Pro, anti, pro/anti

These tests were designed to assess basic visuomotor performance, inhibition, and mental flexibility, respectively (Forsyth et al. 2016; White et al. 2018). Participants were instructed to maintain their focus on a white fixation dot (sized 0.3° of visual angle and positioned in the centre of the screen). After a varying delay of 400 ms, 600 ms, 800 ms, 1000 ms or 1200 ms, a 2° square appeared 8° left or right of the fixation dot. These conditions were counterbalanced and randomised. In Pro the square was green and the participant was required to press the button box key positioned on the same side as the square. In Anti the square was red and the participant was required to press the key on the opposite side from the square. Participants were asked to respond as quickly as possible without compromising accuracy. In Pro/Anti, both red and green squares appeared in a random order and the participant responded in accordance to the rules learned in the previous tasks. For all the tasks, if the participant gave an incorrect response, responded in less than 100 ms or did not respond within 1500 ms, a 900 Hz frequency error tone sounded for 300 ms. Each task had 40 experimental trials preceded by practice trials (4 for Pro and Anti, 6 for Pro/Anti). For each of the tasks reaction time (RT) and accuracy were recorded.

##### Forward and backward spatial

These tasks were computerised versions for the Corsi block-tapping tasks, designed to assess spatial short-term memory storage capacity and manipulation, respectively (Ramos et al. 2022; White et al. 2018). At the beginning, nine grey boxes (38 × 34 mm) appeared in a fixed arrangement on the screen. Following a delay of 100 ms, one box at a time turned white for 1000 ms before returning to grey in a predetermined sequence. A 300 ms tone signalled the end of the sequence. The participant used the mouse to click on the boxes in the same order (Forward Spatial) or in the reverse order (Backward Spatial) they had turned white and verbally indicated that they had finished doing so, following which the experimenter began the next sequence. The sequences started with two boxes and could continue up to nine boxes, with each sequence length occurring twice, before increasing in number (e.g., two boxes, two boxes, three boxes, three boxes, etc.). The sequences (Online Resource 2) differed for forward versus backward but were the same across participants and sessions. The participant had to achieve at least one correct sequence of each length to continue (e.g., an incorrect recall of two boxes followed by a correct recall of two boxes would allow the task to continue), otherwise the task ended. For both Forward and Backward Spatial, participants completed two practice sequences of two boxes before attempting to complete up to 18 test sequences. For these tasks, participants were asked to respond as accurately as possible and were told that speed was not recorded.

##### Forward and backward digit

These tasks were designed to test verbal short-term memory storage capacity and manipulation, respectively (Kouwenhoven and Machado 2023; White et al. 2019a, b) and followed the same procedure as Forward and Backward Spatial, with the following exceptions. Participants were cued with a 0.3° white fixation dot for 500 ms before being shown a sequence of numbers (font: Liberation Sans; size: 50), after which the tone sounded and the fixation dot was shown again for 500 ms, followed by a blank screen. Participants responded verbally, recalling the numbers they had been shown in the order specified (forward or backward). The researcher marked the response as correct or incorrect by pressing the “C” or “X” key. The number sequences differed for forward versus backward but were the same across all participants and sessions.

#### Enjoyment visual analogue scale

A visual analogue scale (Machado et al. 2019) assessed how much the runners enjoyed engaging in the running regime assigned to them. Runners viewed the instruction “*Click the position on the line that best represents how much you enjoyed the running regime.”* Beneath this was a 100 mm line with 101 clickable points, corresponding to scores ranging from 0 to 100; runners clicked on the line using a mouse to indicate their level of enjoyment from “*not at all”* to “*extremely*”; they could readjust their response until they clicked a button labelled “*done*”.

#### Fitness test

Submaximal cardiorespiratory fitness was measured via the YMCA 3-min step test, which has previously been validated as an adequate measure of aerobic capacity (Beutner et al. 2015; Castro-Piñero et al. 2021; Hong et al. 2019; Kieu et al. 2020), and although the literature largely lacks relevant retest reliability data (Kieu et al. 2020) the limited available data indicates excellent reliability (McArdle et al. 1972). The test required participants to step up and down 24 times on a 30 cm step for a period of 3 min at a rhythm of 96 beats per minute which was aided by the Soundbrenner metronome app (https://www.soundbrenner.com/the-metronome-app). The participant was allowed to practice the motion a few times before the start of the test. For their safety, participants were told to put their entire foot on the step to ensure stability and were reminded of this during the test. Immediately following the end of the 3 min, the participant was asked to sit down and their heart rate was recorded for a period of 1 min. Average heart rate and maximum heart rate during and after the fitness test (which provided objective measures of fitness) were collected. To improve measurement accuracy, the participants’ date of birth, height, weight and sex were put into the watch prior to the beginning of the fitness test. Estimated VO_2MAX_ was calculated using the Eq. (1) from Beutner et al. (2015). In accordance with their protocol, heart beat count (HBC) was calculated by averaging the heart rates (recorded by the heart rate watch) every 5 s during the 1 min recovery period.

1 $$ {\text{Est}}.{\text{VO}}_{{{\text{2MAX}}}} = {78}.{2}{-}\left( {0.{38}*{\text{age}}} \right){-}\left( {{4}.{2}*{\text{sex}}\left[ {{\text{male}} = {2},{\text{female}} = {1}} \right]} \right){-}\left( {0.{15}*{\text{HBC}}} \right) $$

#### Record of exercise

Each week all participants received an electronic word document record of exercise (Online Resource 3), in which they were asked to record details of any exercise (e.g., the required running and any additional exercise sessions) that elevated their heart and breathing rate. It included details such as the date, time of day, distance, duration and details of the exercise including environmental conditions. Runners were instructed to include details of any missed running sessions and verbally instructed not to record PA associated with standard daily activities (e.g., walking to and from university or work).

### Statistical analysis

The responses from the NZ-PAQ were used to calculate the amount of PA (h/week). These were compared across time points and groups using paired-samples and independent-samples *t* tests respectively, as were demographic characteristics (e.g., age and education) as well as physiological characteristics (e.g., BMI); note that independent-samples *t* tests were needed to specifically test for group difference at baseline. A chi-squared test was used to compare baseline differences between groups on sex, handedness and ethnicity. Shapiro–Wilk tests assessed all of the data for normality.

RTs were the main variable of interest for Pro, Anti and Pro/Anti, since we anticipated ceiling accuracy rates (White et al. 2018, 2022); to reduce the effects of outliers, median RT was analysed for each participant. For the Digit and Spatial tasks, total score (i.e., the product of the longest sequence length achieved and the total number of sequences recalled correctly) was used as the main variable of interest. For positive and negative affect, the sums for each measure were calculated and used as the main variables of interest. For the main analysis of cognitive and affect scores, mixed model repeated-measures analyses of covariance (RM ANCOVA) were used with time (time 1 [T1] or time 2 [T2]) as the within subjects variable and group (runner or control) as the between subjects variable. Sex, age, education and handedness were included as covariates (note that we did not have enough power to consider these as explanatory variables).

Participants’ estimated VO_2MAX_ were used in a mixed model RM ANCOVA to assess whether the 6-week exercise regime had improved the runner group fitness compared to the control group between T1 and T2. A RM analysis of variance (ANOVA) was subsequently performed on subsamples of the participants to account for poor adherence in the runners and non-sedentary controls. We calculated intraclass correlation coefficients (ICCs) to estimate the retest reliability of the fitness test using a 2-way mixed effect model with absolute agreement, based on a mean rating (K = 8). Participant fitness was compared to normative fitness data (Riebe et al. 2018) for males and females age 20–29 years.

Environment was determined using the responses from the Records of Exercise regarding the total number of running sessions completed either outdoors or indoors. Runners were then grouped based on the environment they exercised in most (i.e., indoors or outdoors). A mixed model RM ANCOVA was used to assess the influence of environment on changes in cognitive, affect and fitness scores. Also within the runner group, Pearson’s correlations assessed relationships between the level of enjoyment of the running regime and change scores for each neuropsychological measure and fitness, as well as running adherence. Follow-up *t* tests were used to investigate any significant or medium-to-large-sized interactions; in cases of medium-to-large interactions that did not reach statistical significance, Bayes factors (BF_10_) were reported to assist in the interpretation.

Although ANCOVAs have been found to be robust to violations of normality (Chen et al. 2019), a generalised linear mixed model (GLMM) analysis was conducted on the neuropsychological and fitness T1 and T2 data to confirm the ANCOVA results given the small sample size (which resulted in a large amount of variation) and unbalanced group sizes (due to the 3:1 allocation ratio). If the data were found to be non-normal following a Shapiro–Wilk test, outliers were removed to assess whether they were causing the non-normal distribution. In the case that they were, the GLMM was run with a linear distribution and an identity link. If the removal of the outliers did not resolve the normality issue, the GLMM was run using a gamma distribution and a log link with the outliers retained in the sample in order to conserve power. Group, Time and the covariates in the ANCOVA were fixed effects, whereas subject ID was a random effect.

All analyses were run using Statistical Package for the Social Sciences (SPSS), except BF_10_ were calculated using R Studio (https://www.rstudio.com/) default settings for both the *t* tests (noninformative Jeffreys prior placed on the variance of the normal population, scale of 0.707, Cauchy prior placed on the standardized effect size) and correlations (noninformative priors assumed for population means and variances, scaled beta prior distribution of medium width assumed). Prior to the start of the trial, a power analysis was run using G*Power (Faul et al. 2007), which indicated that with the desired power level of 80%, 20 participants (10 runners and 10 controls) would be sufficient to detect any repeated measures ANCOVA group x time interactions given an effect size of *ƞp*^2^ = 0.23, which could be expected based on the group x time interaction for the visuospatial memory measure in Stroth et al. (2009); this effect size sits between the effect sizes indicated by the group x time interactions for the positive affect measure (*ƞp*^2^ = 0.11) and the fitness measure (*ƞp*^2^ = 0.33) in Stroth et al. (2009). Statistical significance was based on a *p* < 0.05 and effect sizes are reported as partial eta squared (*ƞp*^2^) for the ANCOVAs and ANOVAs, Hedges’ *g*_*av*_ for the *t* tests, *r* for the Pearson’s correlations, and phi coefficient (φ) for the chi-squared tests. Hedges’ *g*_*av*_ has been recommended as a more appropriate effect size to be used when dealing with small sample sizes (Ramos and Machado 2021) and the phi coefficient acted as effect size for the chi-square test as recommended when 2 × 2 contingency tables are used (Kotrlik et al. 2011). Effect sizes were interpreted as small (*ƞp*^*2*^ = 0.01, *g* = 0.20, φ = 0.10, *r* = 0.10), medium (*ƞp*^*2*^ = 0.06, *g* = 0.50, φ = 0.30, *r* = 0.30) or large (*ƞp*^*2*^ = 0.14, *g* = 0.80, φ = 0.50, *r* = 0.50) (Cohen 1988). Considering recommendations against overreliance on *p*-values and that effect sizes should also be taken as meaningful (Andrade 2019; Wasserstein and Lazar 2016; Wasserstein et al. 2019), we interpreted at least medium effect sizes (*ƞp*^*2*^ = 0.06, *g* = 0.50, φ = 0.30, *r* = 0.30) as results of interest (Nasrollahi et al. 2022) and included BF_10_ to aid with interpretation of *t* test results, using recommended benchmarks (see Fig. 1 in Kelter 2020).

## Results

The data were first examined for the following performance criteria: above 60% accuracy (Pro, Anti, Pro/Anti) and a minimum span of three (Spatial and Digit tasks). These criteria led to the backward digit data being removed for one runner due to a span of only two being achieved at T1. Table 1 summarises the demographic variables for each group (age ranges: controls 18–22 years and runners 18–28 years; education ranges: controls 12–15 years and runners 11–21 years) and shows no significant group differences, although age showed a medium effect size in the direction of the runner group being older. Both groups identified mainly as NZ European (see Table 1), with the next largest contingent identifying as Māori and another ethnicity (NZ European, Samoan or Pakistani; runner 12.5% and control 0%). Participants also identified as Sri Lankan (*n* = 1), Iraqi (*n* = 1), Australian European (*n* = 1), Singaporean Chinese *(n* = 1), Cook Islands Māori/Tongan/Niuean (*n* = 1) and Israeli (*n* = 1).

**Table 1:** Demographic statistics for each group (runner and control) and group difference statistics

Demographic characteristics	Runner (N = 24)	Control (N = 8)	*p*-value	Effect size
Age (mean years)	20.71 (2.76)	19.13 (1.27)	0.139	0.60 (*g*)
Right-handed (%)	95.83	87.50	0.399	0.15 (φ)
Male (%)	20.83	25.00	0.807	0.04 (φ)
New Zealand European (%)	66.67	87.50	0.649	0.28 (φ)
Education (mean years)	14.10 (1.93)	13.63 (0.86)	0.514	0.26 (*g*)

Standard deviations in parentheses. *g* = Hedges’ *g*_*av*_ (0.20 = small, 0.50 = medium, 0.80 = large) and φ = Phi coefficient (0.10 = small, 0.30 = medium, 0.50 = large)

Table 2 summarises by group at T1 and T2 results from the fitness test, NZ-PAQ, BMI, cognitive tasks, PANAS and, for the runner group only, running enjoyment and adherence to the running. Table 3 summarises by group change scores for the measures as well as group x time interaction test statistics,* p*-values and effect sizes.

**Table 2:** Runner group and control group BMI, NZ-PAQ, fitness, cognitive, affect, enjoyment and adherence measures at Time 1 and Time 2

Measure	Runner (N = 24)			Control (N = 8)			
	Time 1	Time 2	Corr	Time 1		Time 2	Corr
BMI (kg/m^2^)	25.33 (5.62)	25.15 (5.55)	.991	23.10 (2.92)		23.33 (2.84)	0.990
NZ-PAQ (h/week)	3.53 (3.56)	4.14 (3.51)	.391	2.41 (2.90)		3.60 (4.96)	.819
VO_2MAX_ (ml/kg/min)	44.21 (3.78)	43.67 (3.86)	.700	45.65 (4.74)		44.88 (4.29)	.955
Pro (ms)	302 (33.43)	301 (51.44)	.586	280 (22.27)		284 (20.13)	.681
Anti (ms)	358 (56.62)	351 (50.51)	.725	340 (34.52)		330 (27.79)	.647
Pro/Anti (ms)	523 (76.81)	483 (89.40)	.797	490 (85.62)		463 (82.24)	.917
Forward Spatial	56.79 (18.90)	63.5 (20.23)	.641	60.63 (17.52)		66.63 (15.14)	.746
Backward Spatial	54.54 (22.56)	60.58 (20.97)	.254	60.00 (13.19)		50.75 (19.41)	− .023
Forward Digit	58.63 (19.20)	62.00 (23.57)	.202	47.13 (14.37)		55.00 (29.14)	.551
Backward Digit	35.52 (12.38)^†^	39.61 (12.55)^†^	.346	38.88 (11.26)		37.63 (20.14)	.505
Positive Affect	30.67 (6.36)	31.63 (5.80)	.554	32.63 (4.03)		33.75 (5.70)	.786
Negative Affect	18.42 (4.86)	17.17 (5.15)	.662	19.38 (5.17)		16.50 (3.84)	.312
Running Enjoyment	–	61.83 (17.17)	–	–		–	–
Running Adherence	–	15.78 (3.57)	–	–		–	–

Means with SD in parentheses and correlations between Time 1 and Time 2 (see columns labelled Corr). Adherence refers to the runner group’s mean total reported completed runs out of 18 prescribed. The visual analogue enjoyment scale ranged from 0 to 100

*ms* millisecond

^†^*n* = 23

**Table 3:** Change scores for the runner group and control group with interaction test statistics, *p*-values and effect sizes for RM ANCOVA interactions

Measure	Runner (N = 24)	Control (N = 8)	F-statistic	*p*-value	Effect size
VO_2MAX_ (ml/kg/min)	− 0.54	− 0.77	0.11	.746	0.004
Pro (ms)	− 0.33	4.56	0.41	.525	0.016
Anti (ms)	− 7.01	− 9.44	0.06	.807	0.002
Pro/Anti (ms)	− 39.46	− 27.02	0.41	.527	0.016
Forward Spatial	6.71	6.00	0.04	.837	0.002
Backward Spatial	6.04	− 9.25	0.86	.362	0.032
Forward Digit	3.38	7.88	0.03	.854	0.001
Backward Digit	3.92^†^	− 1.25	1.52	.229	0.057
Positive Affect	0.96	1.13	0.07	.793	0.003
Negative Affect	− 1.25	− 2.88	0.73	.400	0.027

Effect sizes are partial eta squared (small = 0.01, medium = 0.06, large = 0.14). For Pro, Anti, Pro/Anti and Negative Affect, a negative score indicates an improvement. For all other measures, a positive score indicates improvement

^†^*n* = 23

### BMI, NZ-PAQ, and fitness measures

No significant differences were detected between the two groups at either time point for BMI (Time 1, *t*(30) = 1.04, *p* = 0.307, *g* = 0.41, BMI ranges: controls 17.37–28.50 kg/m^2^ and runners 20.13–39.50 kg/m^2^; Time 2, *t*(30) = 0.87, *p* = 0.394, *g* = 0.34, BMI ranges: controls 17.67–28.08 kg/m^2^ and runners 19.92–39.79 kg/m^2^) or NZ-PAQ (Time 1, *t*(30) = 0.78,* p* = 0.441, *g* = 0.31, NZ-PAQ ranges: controls 0.33–9.00 h/week and runners 0.00–12.53 h/week; Time 2,* t*(30) = 0.33, *p* = 0.744, *g* = 0.13, NZ-PAQ ranges: controls 0.00–14.50 h/week and runners 0.17–18.00 h/week). Moreover, from T1 to T2, BMI showed no significant change for the runner group, *t*(23) = -1.17, *p* = 0.255, *g* = − 0.23, or the control group, *t*(7) = 1.42, *p* = 0.198, *g* = 0.48. Regarding the NZ-PAQ, both groups showed a numerical increase in reported PA from T1 to T2, with the average increase numerically larger in the control group (> 1 h/week) than in the runner group (< 1 h/week), but the increases were not statistically significant in either group: control group, *t*(7) = 1.03, *p* = 0.339, *g* = 0.34; runner group, *t*(23) = 0.76, *p* = 0.458, *g* = 0.15.

To assess whether the fitness levels of the participants in the current research appear similar to normative data, the estimated VO_2MAX_ at T1 was compared to data provided by the American College of Sports Medicine (ACSM) (Riebe et al. 2018). The average VO_2MAX_ of the females in the current research (*M* = 43.02) exceeded the average of the normative data (*M* = 37.67), and the range (38.14 – 49.55) sits in the 50th to 90th percentile given by the ACSM for ages 20–29 years. For the males, the average VO_2MAX_ (*M* = 50.09) also exceeded the normative mean (*M* = 47.59), and the range (46.36 – 51.81) sits in the 45th to 75th percentile for ages 20–29 years. Visual examination of the fitness results in Table 2 shows that both groups had a numerically lower VO_2MAX_ after the 6-week intervention period, in contrast to the expectation that fitness would increase in the runners. A 2 (time) × 2 (group) RM ANCOVA with fitness as the dependent variable showed no main effects or interactions. However, fitness showed a between subjects effect of both sex, *F*(1, 26) = 29.77, *p* < 0.001, *ƞp*^*2*^ = 0.53, which reflected the expected pattern that irrespective of time point and group males (T1 *M* = 50.09, T2 *M* = 49.00) had higher VO_2MAX_ than females (T1 *M* = 43.02, T2 *M* = 42.57), and age, *F*(1, 26) = 6.61, *p* = 0.016, *ƞp*^*2*^ = 0.20, which reflected the expected pattern that irrespective of time point and group younger age was associated with higher fitness.

### Neuropsychological measures

Visual examination of the cognitive change scores in Table 3 provides limited numerical indications of greater improvements in the runners than the controls. RM ANCOVAs found no significant or medium-sized main effects or interactions for any of the cognitive measures. With regard to covariates, Forward Spatial showed a significant effect of sex, *F*(1, 26) = 6.59, *p* = 0.016, *ƞp*^*2*^ = 0.20, such that males performed worse than females. Forward Digit showed a significant time x sex interaction, *F*(1, 26) = 8.13, *p* = 0.008, *ƞp*^*2*^ = 0.24, reflecting a tendency for male but not female Forward Digit scores to improve from T1 to T2. Care should be taken in interpreting these sex effects beyond the current sample given that it included a total of only seven males.

Visual examination of the affect data in Table 2 indicates that both groups appear to have improved slightly in both positive and negative affect scores (i.e., numerically more positive and less negative at T2). However, 2 (time) × 2 (group) RM ANCOVAs found no significant or medium-sized main effects or interactions for either positive or negative affect, and the changes from T1 to T2 were numerically smaller in the runner group (see Table 3).

### Confirmation analysis

GLMMs analysed the fitness, cognitive, and affect data to confirm the results of the ANCOVAs. All results were in line with the initial models (such that no statistically significant interactions or main effects were observed except with regard to the covariate effects).

### Environment

To investigate if running environment had any effect on outcome variables for the runners, we ran a 2 (time) × 2 (environment) RM ANOVA that included runners categorised as indoor (*n* = 15) or outdoor (*n* = 6). Note that we excluded two runners for failing to return a full set of records and one runner due to running half indoors and half outdoors, leaving 21 runners in the environment analysis.

Change scores and interaction effect sizes can be found in Table S1 (Online Resource 4). A large, albeit non-significant, time x environment interaction was found for Forward Spatial score, *F*(1, 19) = 3.54, *p* = 0.075, *ƞp*^*2*^ = 0.16. Exploratory follow-up paired-samples *t* tests found for indoor runners a small non-significant improvement (T1 *M* = 58.07, T2 *M* = 62.27), *t*(14) = 1.13, *p* = 0.279, *g* = 0.28, BF_10_ = 0.450, with BF_10_ indicating anecdotal evidence for the null hypothesis. In contrast, outdoor runners showed a large non-significant improvement in Forward Spatial scores (T1 *M* = 50.67, T2 *M* = 69.67), *t*(5) = 2.26, *p* = 0.074, *g* = 0.85, BF_10_ = 1.581, with BF_10_ indicating anecdotal evidence for the alternative hypothesis. Another large non-significant time x environment interaction was found for negative affect, *F*(1, 19) = 4.04, *p* = 0.059, *ƞp*^*2*^ = 0.18. Negative affect for indoor runners was similar across the time points, *t*(14) =  − 0.14, *p* = 0.894, *g* =  − 0.03, BF_10_ = 0.265, with BF_10_ indicating moderate evidence for the null hypothesis. In contrast, there was a large non-significant improvement in negative affect for outdoor runners, *t*(5) = - 2.19, *p* = 0.080, *g* =  − 0.83, BF_10_ = 1.488, with BF_10_ indicating anecdotal evidence for the alternative hypothesis. Outdoor runners began with a numerically higher negative affect score (*M* = 19.00) than indoor runners (*M* = 16.40), which lowered such that at T2 outdoor runners’ negative affect score (*M* = 15.00) was numerically lower than the indoor runners’ score (*M* = 16.27). Care should be taken in interpreting these exploratory results given the small number of outdoor runners.

### Enjoyment

Running enjoyment scores ranged from 32 to 91 (scale range: 0–100). Pearson’s correlations evaluated relationships between enjoyment of the exercise regime and change scores for the fitness, cognitive and affect measures as well as running adherence (see Table S2, Online Resource 4). The sample size was the same in all cases (*n* = 24) except for in Backward Digit (*n* = 23). A large-sized significant negative relationship was observed between enjoyment and Forward Digit change score, *r*(24) = − 0.58, *p* = 0.003, BF_10_ = 15.842, such that higher levels of running enjoyment were associated with a greater drop in performance on the Forward Digit task (see Figure S1 in Online Resource 4); BF_10_ indicated strong evidence for the alternative hypothesis. A non-significant medium-sized effect was found between enjoyment and change in positive affect, *r*(24) = 0.34, *p* = 0.105, BF_10_ = 1.290, such that higher levels of running enjoyment were associated with greater increases in positive affect scores; BF_10_ indicated anecdotal evidence for the alternative hypothesis. No other significant or at least medium-sized effects were found.

### Subsample fitness analysis

As runners varied quite substantially in reported adherence (5–18 total runs completed) and not all controls maintained a sedentary regime, we conducted a secondary analysis of changes in fitness in a subsample of participants. Runners were excluded if they reported completing less than 16 of the prescribed 18 runs (*n* = 7), failed to return a full set of records of exercise (*n* = 2), or if their T1 NZ-PAQ responses indicated they were, in contrast to their claim when screened, more physically active than allowed (*n* = 2). This left a total of 13 runners. One control participant was excluded after visual examination of their NZ-PAQ data indicated a substantial increase in PA from T1 to T2 (T1 = 5.25 h, T2 = 14.50 h), contrary to the requirement to maintain their current level of physical activity. This left seven controls (including two controls who showed smaller increases in PA from T1 to T2: T1 = 1.25 h, T2 = 1.83 h and T1 = 1.43 h, T2 = 2.20 h) and a total of 20 participants in the analysis. Table 4 summarises the subsample’s fitness and NZ-PAQ data at T1 and T2 as well as mean adherence amongst the runners. Surprisingly, we again found no evidence that fitness increased from T1 to T2 in the runner subsample and a 2 (time) × 2 (group) RM ANOVA run on the fitness data showed no significant main effects or an interaction: Group, *F*(1, 18) = 1.08, *p* = 0.313, *ƞp*^*2*^ = 0.06; Time, *F*(1, 18) = 0.56, *p* = 0.464, *ƞp*^*2*^ = 0.03; interaction, *F*(1, 18) = 0.26, *p* = 0.617. *ƞp*^*2*^ = 0.01.

**Table 4:** Subsample runner and control group NZ-PAQ and fitness and runner adherence

Measure	Runner (N = 13)			Control (N = 7)		
	Time 1	Time 2	Corr	Time 1	Time 2	Corr
NZ-PAQ (h/week)	2.93 (2.77)	4.90 (4.32)	.414	2.00 (3.11)	2.04 (3.18)	.990
VO_2MAX_ (mg/kg/min)	43.90 (4.64)	43.72 (4.11)	.677	46.34 (5.06)	45.44 (4.64)	.949
Adherence	–	17.62 (0.87)	–	–	–	–

Means with SD in parentheses and correlations between Time 1 and Time 2 (see columns labelled Corr). Adherence is given as mean total number of runs

### Reliability of the YMCA 3-min step test

An ICC was calculated to assess the retest reliability of the YMCA 3-min step test using the control participants’ data (*n* = 8). A high amount of reliability was found between the results of the fitness test at T1 and T2, with an ICC = 0.971 and 95% CI between 0.864 and 0.994.

## Discussion

This study aimed to advance our understanding of the potential benefits of regular, long-term exercise on the cognitive function and affect of sedentary (i.e., not exercising more than twice a week) but healthy young adults via an intervention trial in which university students randomised into an exercise group were asked to run for 30 min three times a week for 6 weeks (based on Stroth et al. 2009). Unexpectedly, the running intervention failed to induce improvements in the majority of the measures, including fitness. Setting aside this major limitation, a few results met our threshold for interpretation. Namely, greater enjoyment of the running regime had a medium-sized association with larger increases in positive affect. In addition, an unexpected negative correlation was found between enjoyment and Forward Digit change scores. Also, with regard to environment, outdoor runners showed large-sized improvement effects for both Forward Spatial score and negative affect. While some of the enjoyment and environment results point towards interesting avenues for further research, the lack of benefits of the running intervention for fitness and generally for neuropsychological function was disappointing. The main contributing factor appears to stem from insufficient increases in PA (both in terms of amount and intensity). This issue and other factors limiting the strength of the outcomes from the current pre-registered randomised controlled trial, including reliance on self-reported adherence and the timing of the trial overlapping with the COVID-19 pandemic, are discussed followed by consideration of the patterns that emerged related to enjoyment and environment and recommendations relevant to future research.

Surprisingly, the runner group did not even show numerical increases in fitness, which suggests that increases in PA must have been quite limited, leaving the lack of improvements in cognitive and affect scores unsurprising. Our findings contrast with previous findings in a similar population that a running intervention improved visuospatial memory, cognitive control and flexibility as well as positive affect (Stroth et al. 2009, 2010), but in these studies fitness significantly improved in the runners. As much of the previous literature has suggested that changes to fitness drive neuropsychological improvements (e.g., Padilla et al. 2014; Silverman and Deuster 2014), the lack of fitness improvements in the current study may explain the lack of neuropsychological improvements in the main analyses, noting also that fitness provided our only objective indicator of increased PA levels, as participants self-reported PA engagement (prescribed runs and other PA).

There are a number of potential reasons why fitness did not improve for the runner group. Of note, the lack of monitoring during exercise sessions prevented participant-specific feedback related to intensity (although we instructed runners to maintain a pace that increased their heart and breathing rates, this clearly did not lead to sufficient intensity to increase fitness). Moreover, lack of monitoring left us relying solely on self-report with regard to running adherence and other PA engagement. It is possible that this led to inaccurate data as previous research has found that under self-report conditions, participants overestimate their level of PA engagement (Brenner and DeLamater 2014) and underestimate their time spent sedentary (Prince et al. 2020). Although in the current study this did not seem to pose an issue at the group level (as the NZ-PAQ data for the runner group did not show increased PA time beyond what would have been expected based on the prescribed runs), at an individual level, the NZ-PAQ data varied fairly wildly from what one would expect based on the trial instructions (e.g., one runner indicated increasing PA by over 13 h/week and eight runners indicated reductions in PA). The latter examples suggest that many runners replaced pre-trial PA with the prescribed runs, undershooting the total amount of PA at intake. Collectively, these factors can explain the lack of fitness improvements. Moreover, the fact that large increases in reported PA did not necessarily coincide with increases in fitness (e.g., one runner indicated increasing PA by 4.67 h/week but fitness dropped) calls into question the accuracy of the NZ-PAQ data.

Furthermore, data from the NZ-PAQ indicated most participants were exercising in excess of 2.5 h per week at intake (despite meeting the sedentary criterion of not exercising more than twice a week). This fits with the fact that the majority of participants laid above the 50th percentile (based on normative VO_2MAX_ data provided by the ACSM) and signals that our sedentary criterion should have included time engaging in PA in addition to frequency. However, it should be noted that Stroth et al. (2009) observed an increase in fitness using a similar exercise regime without any requirement of being sedentary and they also allowed participants to fast walk (similar to the current study), suggesting that the exercise regime in the current study should have been sufficient to drive an increase in fitness. However, in Stroth et al. (2009), the participants were monitored during their exercise sessions and given instructions to exercise to a prescribed intensity level based on heart rate and lactate threshold, which was not the case in the current study. These differences likely underpin fitness improving in Stroth et al. (2009) but not in the current trial. Although intensive monitoring does not suit intervention trials designed with translation to everyday contexts in mind, some level of monitoring and individualized feedback may be necessary, at least in fitter-than-typical participants.

Regarding running enjoyment, our results indicated that higher levels of enjoyment tended to be associated with greater increases in positive affect, which aligns with previous studies reporting a positive association between exercise enjoyment and affect (Greene et al. 2018; Raedeke 2007). However, it should be noted that the association in our study did not reach statistical significance and Bayes factor only indicated anecdotal supportive evidence. Surprisingly, we observed a significant negative correlation between enjoyment and Forward Digit task performance, such that higher enjoyment of the running regime was associated with poorer task performance, which opposes the predicted positive association. Moreover, the predicted positive association was expected to arise through an increase in adherence (Bartlett et al. 2011), however in the current study higher enjoyment levels were only weakly associated with higher adherence. Due to these inconsistencies, we suspect that the negative correlation between enjoyment and Forward Digit performance was spurious.

Regarding past reports that more time in natural environments, including while exercising, can be beneficial to neuropsychological performance (Berman et al. 2008; White et al. 2019a, b), our results suggest that running outdoors may have had some benefit to negative affect and visuospatial short-term memory storage capacity. However, it should be noted that these results did not reach statistical significance, Bayes factor only indicated anecdotal supportive evidence, and only six of our runners met the outdoor criterion (which was based on the predominant running environment; in contrast, all running in the 2009 and 2010 Stroth studies occurred outdoors). Notwithstanding, the large effect sizes indicate that follow-up research involving a larger sample of outdoor runners may be warranted. Given the lack of improvements in fitness, the apparent improvement in Forward Spatial scores for the outdoor group may have related to the Attention Restoration Theory, which posits that cognitive components tapping into directed attention (e.g., working memory) should show the most benefit (Kaplan and Berman 2010). In relation to this, the outdoor runners time spent outside may have been cognitively restorative for their visuospatial short-term memory storage capacity, although it is unclear why performance did not improve for other cognitive tasks, especially more challenging ones, which the theory stipulates should show the most improvement. Regarding the indications of a link between running outdoors, in particular, and reduced negative affect, this finding seems in line with previous findings linking time spent in nature to increased positive affect and higher life satisfaction (Berman et al. 2008; White et al. 2019a, b), although it should be noted that “outdoors” in our study did not exclude city environments. Confirmation of these patterns should ideally involve randomisation into environment groups, with attention paid to the details of the outdoor environment (e.g., a city area or a forest), as some research indicates greater benefits may be gained from more natural outdoor environments (Berman et al. 2008; Wen et al. 2019).

### Limitations and future research

There are a number of limitations of the current randomised controlled trial that must be considered. Of note, reported PA increases (as assessed by the NZ-PAQ) were greater in the controls than the runners, which may reflect controls altering their lifestyles in response to the topic of the study. Although encouraging in the context of the current physical inactivity pandemic (Owen et al. 2010), this signals a need for more intense monitoring if the control group is required to maintain their current level of PA, and suggests that studies aiming to maximise ecological validity may want to consider an active control group in an effort to limit self-determined PA increases. Focusing on the runner group, as previously discussed, adherence to the running regime was based on self-report and so was open to inaccuracies. Accuracy might have been improved by collecting adherence data immediately after the exercise, and adding ratings of perceived exertion could potentially have aided participants to reach sufficient intensity levels to drive fitness increases. Making use of PA monitors or applications on mobile phones could assist on both of these fronts. Mobile applications have previously been found to promote adherence to exercise regimes (Sun et al. 2021), and they have added benefits such as reducing time spent sedentary (van Dantzig et al. 2013) and improving PA and other wellness factors (Du et al. 2016). Moreover, this could enable person-specific intensity targets to be set, thus helping to ensure that fitness increases. However, in terms of translation, it is important to keep in mind that this can lead to the exclusion of low-income individuals, who may be unable to afford the technology required, although the increasing availability of affordable smartphone technology may make this more feasible in the future.

Regarding the fitness test, while the 3-min step test showed excellent retest reliability (albeit with a small sample) and does not appear to be a key limiting factor in the current research, it seems prudent to point out that submaximal tests are not as accurate as maximal tests for assessing fitness (Lee et al. 2019). Thus, it may be advantageous to use a maximal fitness test, as was used in the Stroth et al. (2009, 2010) studies that found fitness improvements, as maximal tests may detect changes to fitness not detected by submaximal tests. Regarding the cognitive variables, retest reliability may have been an issue. Based on White et al. (2018), without prior completion of the tests (i.e., familiarization), Pro/Anti RT showed good reliability, but Pro and Anti RT and spatial scores only showed poor-to-moderate reliability that might have been elevated by including a familiarization session (although that retest reliability study used distinct spatial sequences at the retest points, which may have contributed to the low reliability coefficients, and Pro and Anti only included 20 test trials, in contrast to 40 test trials in the current study). Future intervention trials should take care to confirm that all measures included exhibit adequately high reliability.

The COVID-19 pandemic ongoing throughout the current trial likely imposed a number of influences. For example, lockdown measures enforced in order to control the spread of the virus have recently been found to have a profound attenuating effect on an individual’s PA levels, and this contributed towards lower affect scores (Faulkner et al. 2021; Lopez-Bueno et al. 2020). Barriers to maintaining a regular exercise routine during the COVID-19 pandemic include lack of access to facilities (e.g., for those who normally exercised at a gym) and fear of going outside (Meiring et al. 2021). These findings indicate that runners in the current trial may have found it unusually difficult to engage with the intervention and participants’ neuropsychological scores may have been influenced by the ongoing pandemic in ways not accounted for. Regarding data collection and the sample size, restrictions associated with the pandemic meant people had to stay at home and in-person data collection could not occur during some periods of the trial. Furthermore, the risk of catching COVID-19 may have dissuaded potential participants from being involved in in-person data collection. This presumably contributed towards our study being underpowered (the power analysis indicated a need for at least 10 controls, but there were only eight after exclusions).

The generalisability could be improved by using a more representative sample of young adults, rather than university students (who had free access to exercise facilities) recruited from a primarily female psychology department. One of the motivations behind the current research relates to the concerningly low PA levels amongst young adults, especially females; however, this may have more relevance to young adults working full-time (in contrast to the pattern seen in older adults; Guiney et al. 2018). Targeting sectors of young adults more likely to be sedentary may be a useful advance on the methodology. Moreover, limiting at intake not only the number of days per week of exercise but also the duration per week, and having participants count in their record of exercise PA associated with daily activities (such as active transport), may increase the probability of future interventions successfully increasing participant fitness and with it neuropsychological functioning.

Another limitation of the current study is that we did not document motivation, given previous findings linking higher motivation to exercise with more positive cognitive and behavioural outcomes following an exercise session (Jones et al. 2017). Furthermore, motivation is a determinant of adherence rates (Kohlstedt et al. 2013), thus future studies should document and factor in motivation, and also consider documenting other details of the exercise (such as effort, arousal, and affect) that may be relevant. In addition, allowing choice with regard to modality of exercise could enhance enjoyment by allowing those who do not enjoy running to engage in another exercise modality, and this may come with additional benefits such as increased adherence and greater chance that the individual will continue with the exercise following completion of the trial. Although in the current study mean running enjoyment ratings indicated that on average the runners enjoyed the prescribed exercise, 25% of the runners clicked on the left half of the enjoyment scale, indicating they did not enjoy it very much. Taken together with findings that choosing to exercise leads to greater brain benefits than forced exercise (e.g., Belviranlı and Okudan 2022; Ke et al. 2011; see also Svensson et al. 2016), introducing choice into the design may prove more effective with regard to neuropsychological benefits (note, however, that for some neurological conditions this does not apply; Belviranlı and Okudan 2019; Murray et al. 2014).

## Conclusions

The current study aimed to test whether a running intervention can serve to improve neuropsychological functioning in sedentary, but healthy, young adults. Unfortunately, fitness did not improve in the runners, which likely explains the limited evidence that the running intervention improved cognition or affect. The indications of neuropsychological improvements that did emerge related to time spent outdoors and enjoyment of the running regime. Although somewhat tangential to the focus of the current research, these outcomes point toward potentially fruitful avenues of research. With regard to future research into neuropsychological benefits of chronic exercise, insight gained from the current trial may benefit endeavours to develop effective exercise protocols that can be incorporated into the daily lives of young adults in an effort to shift their physical activity habits from sedentary to active, with the ultimate aim of ensuring that all young adults meet minimum recommended physical activity guidelines.

### Supplementary Information

Below is the link to the electronic supplementary material.

Supplementary file 1

Supplementary file 2

Supplementary file 3

Supplementary file 4

## Data Availability

The dataset analysed during the current study is available upon request from the corresponding author.
