# Supplementary material for: Chronic exercise and neuropsychological function in healthy young adults: a randomised controlled trial investigating a running intervention
Source: Cogn Process. 2024 Feb 29;25(2):241–58. doi: 10.1007/s10339-024-01177-1 (PMC11106121; doi:10.1007/s10339-024-01177-1)

**Online Resource 2**


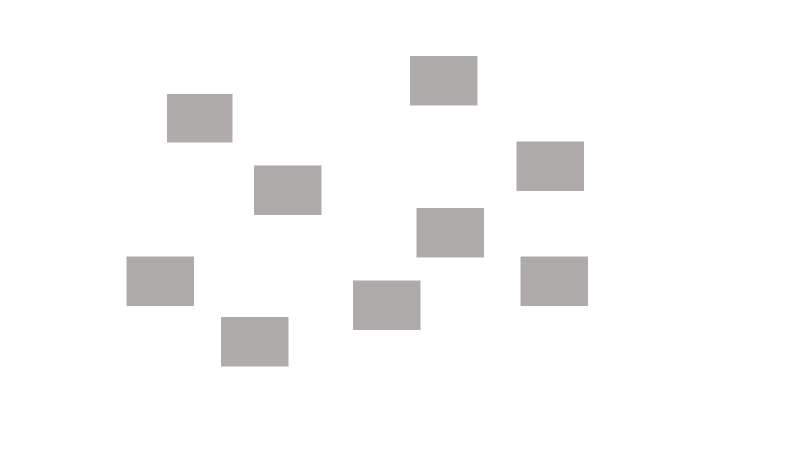
Forward Spatial Task Sequences. The numbers indicate the order in which the boxes turned white. Participants aimed to click the boxes in this order.


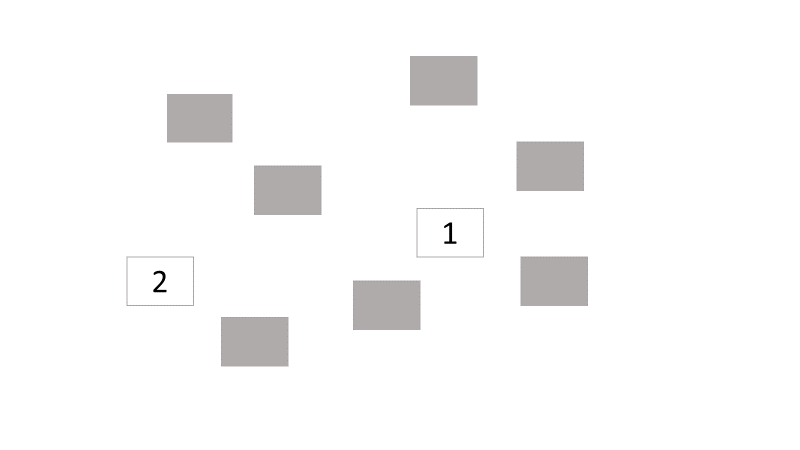


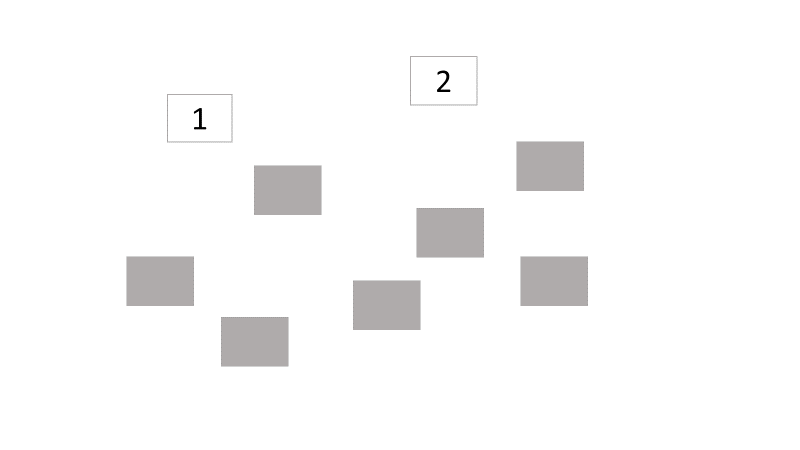


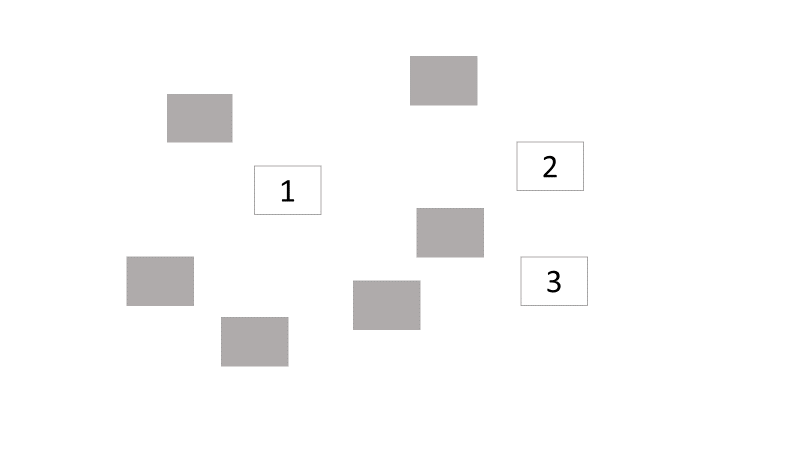


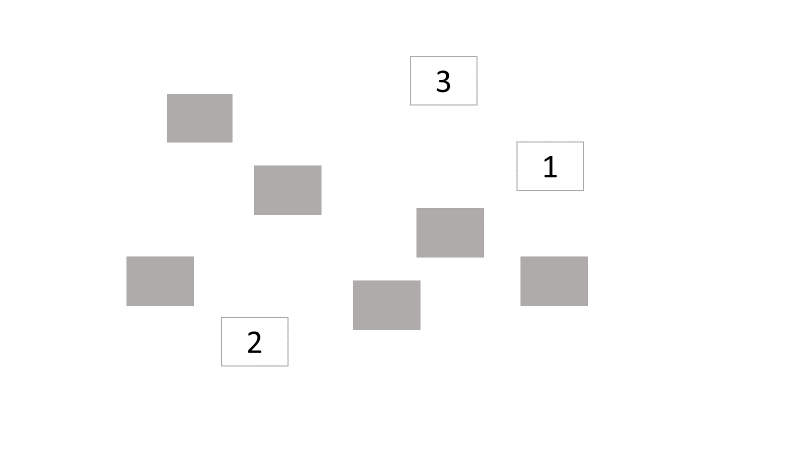

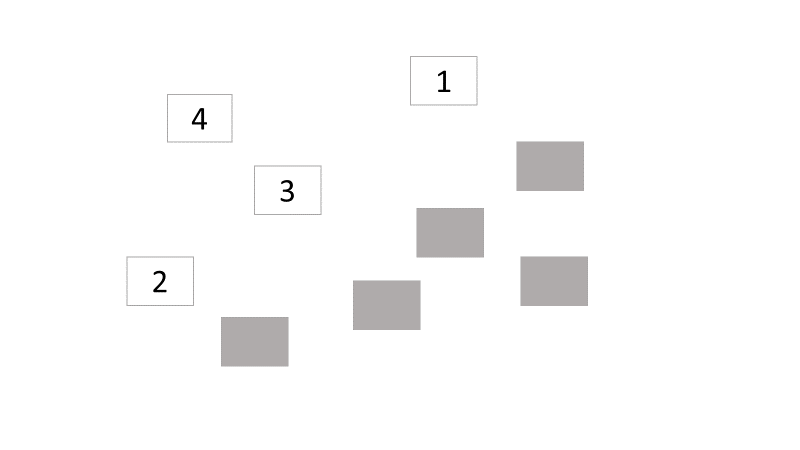


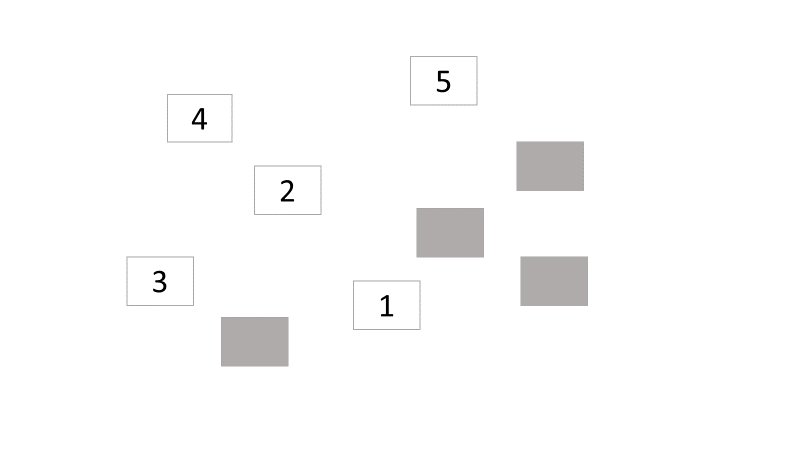


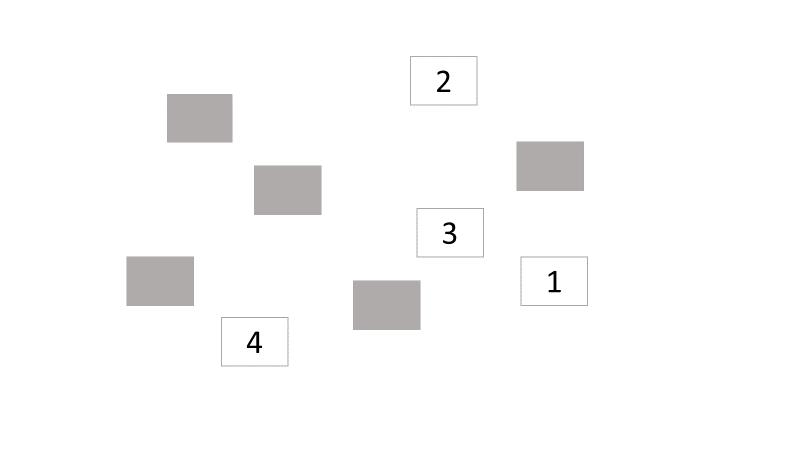


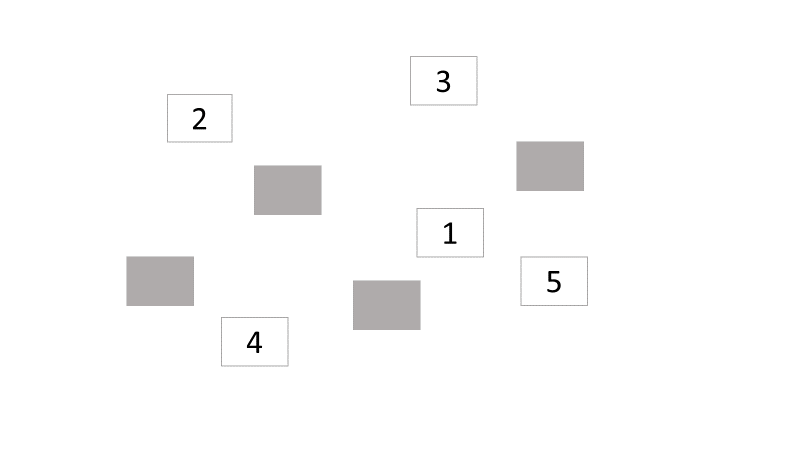


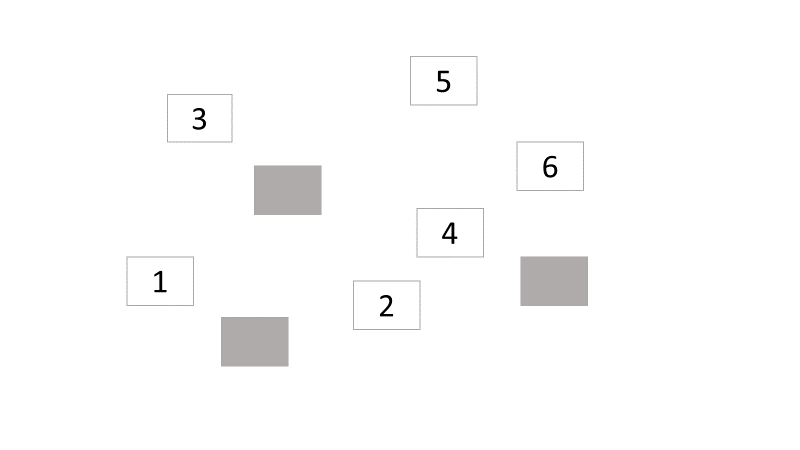


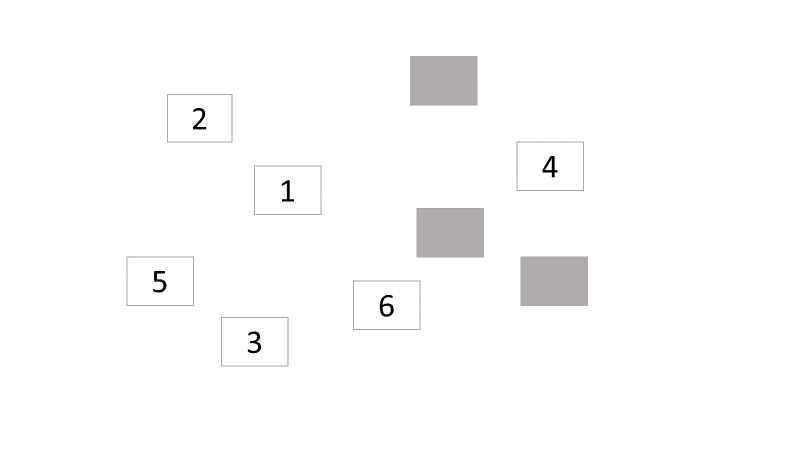


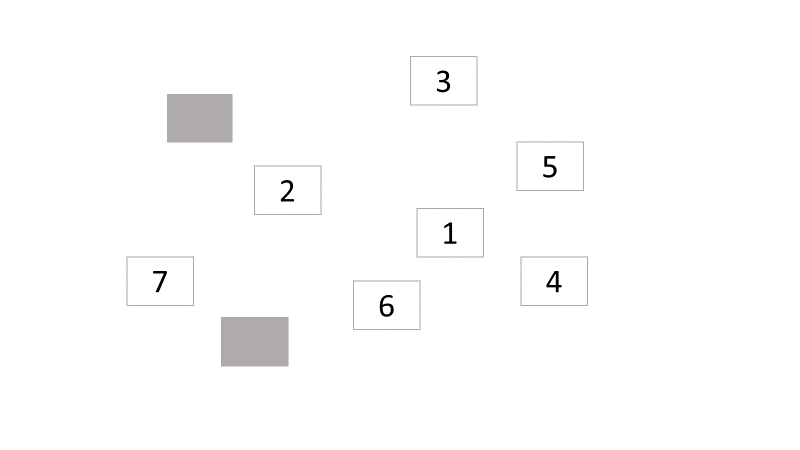


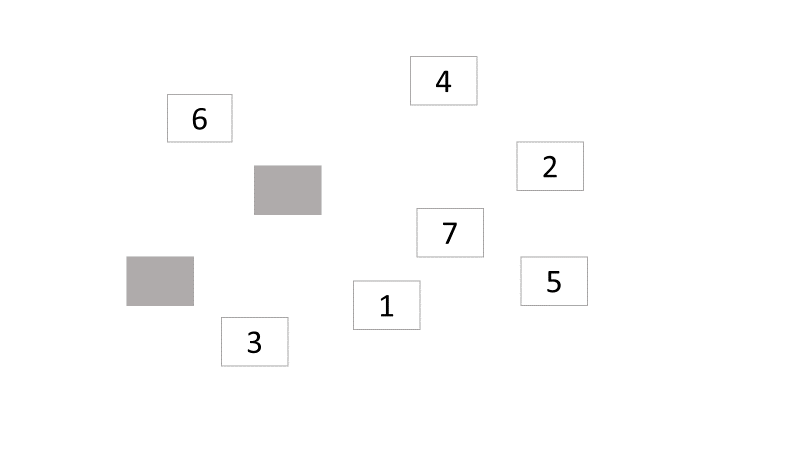


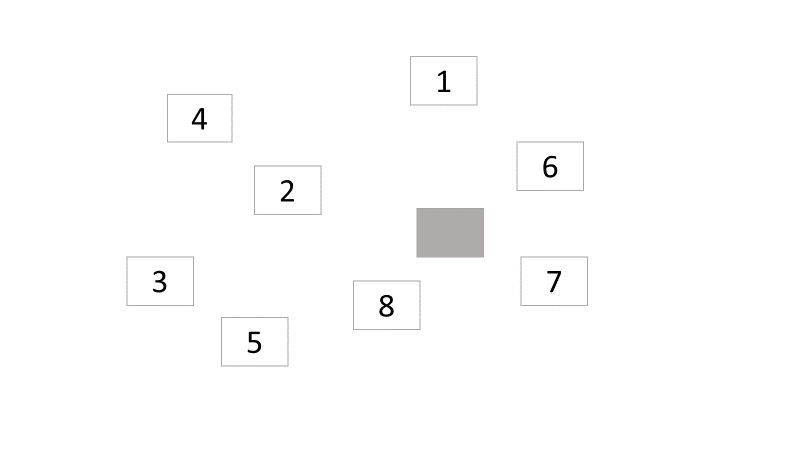


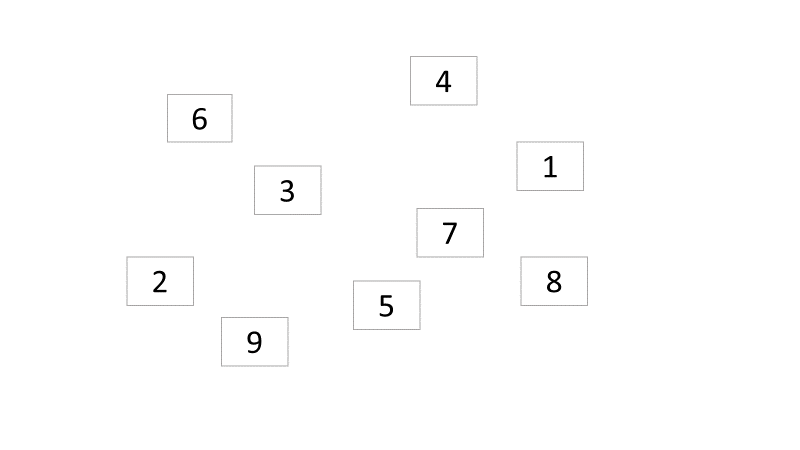


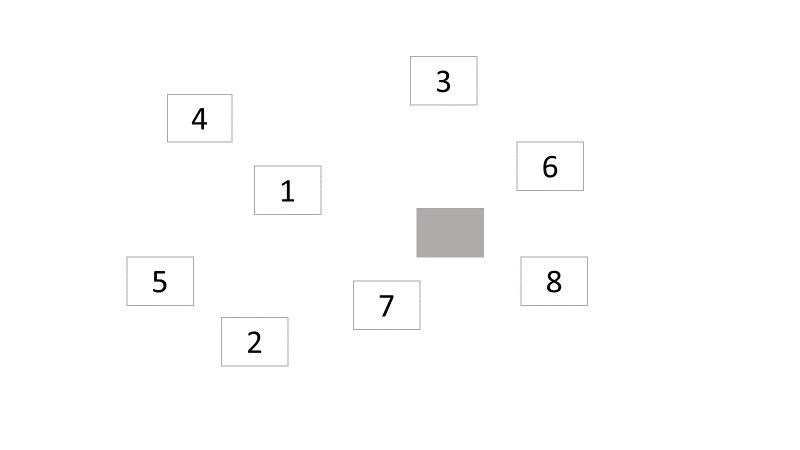


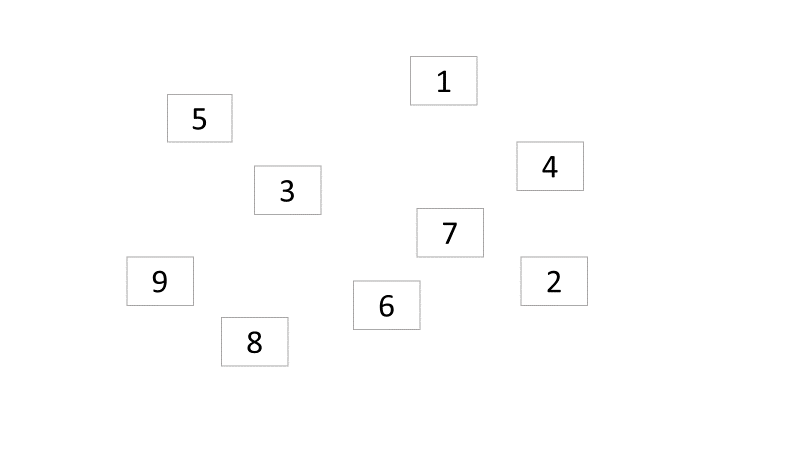


Backward Spatial Task Sequences. The numbers indicate the order in which the boxes turned white. Participant aimed to click the boxes in the reverse order.


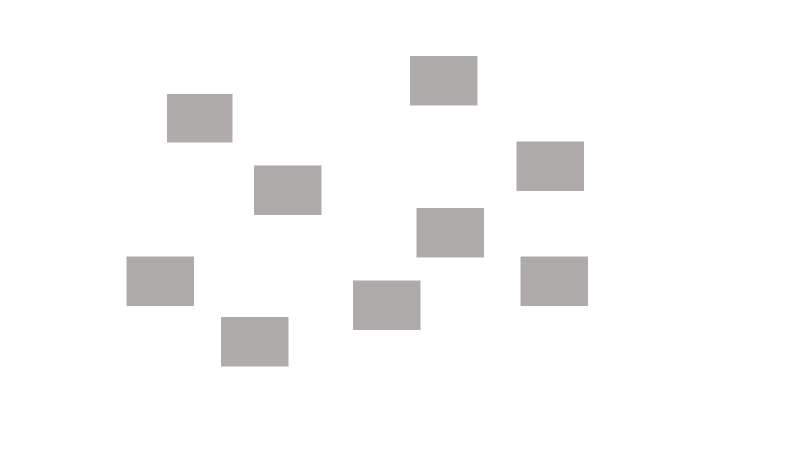


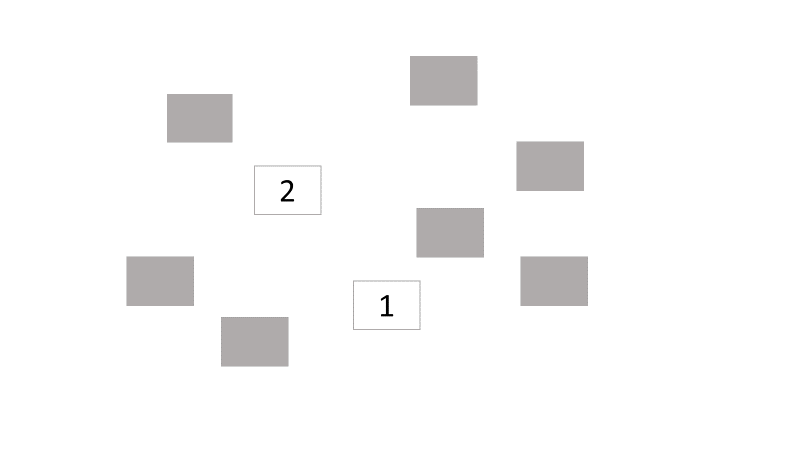


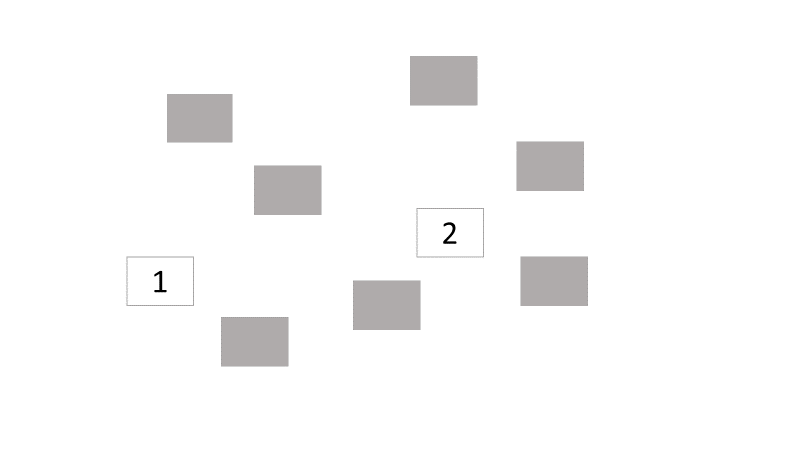


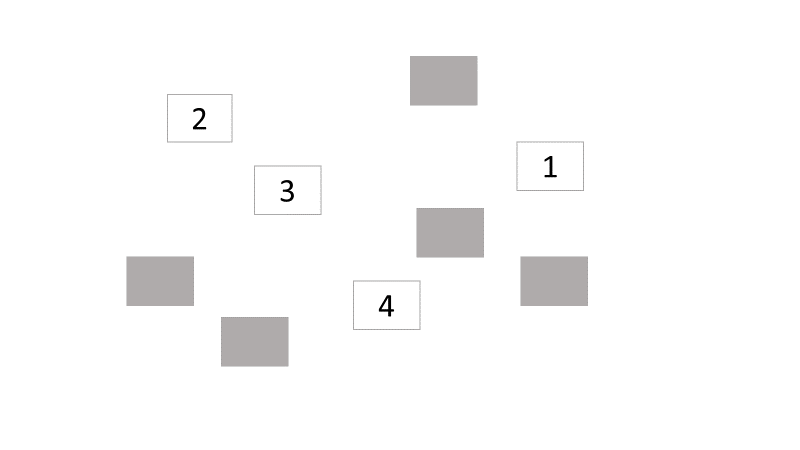

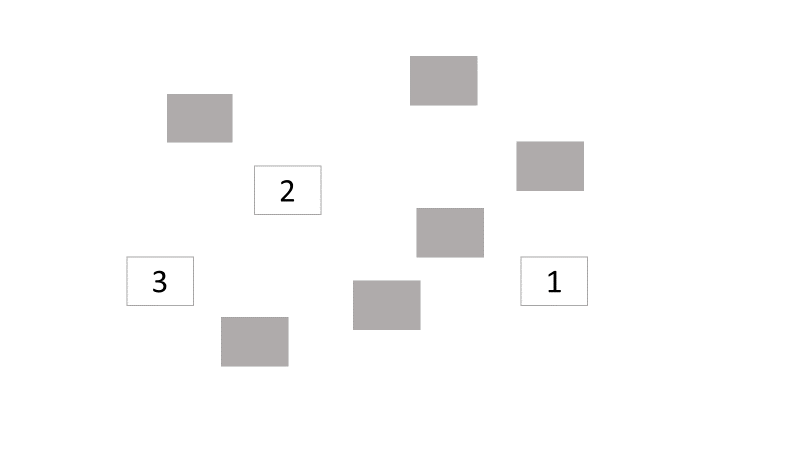


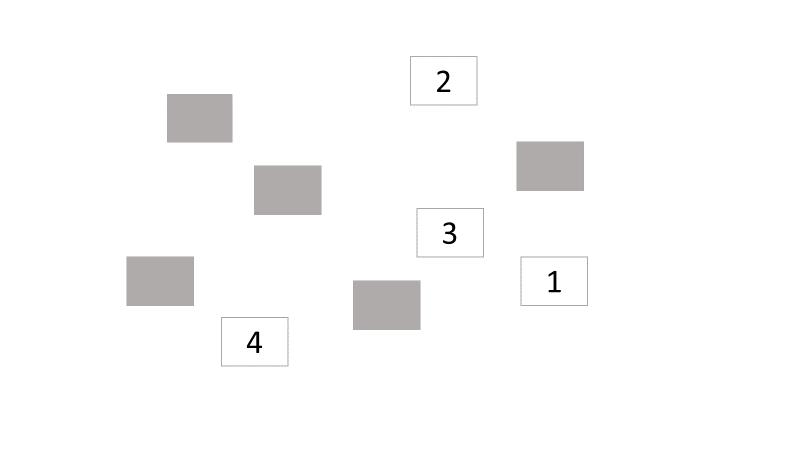

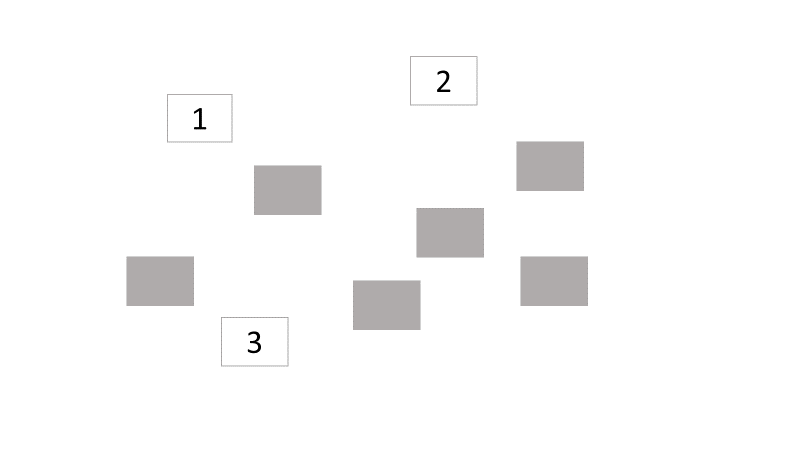


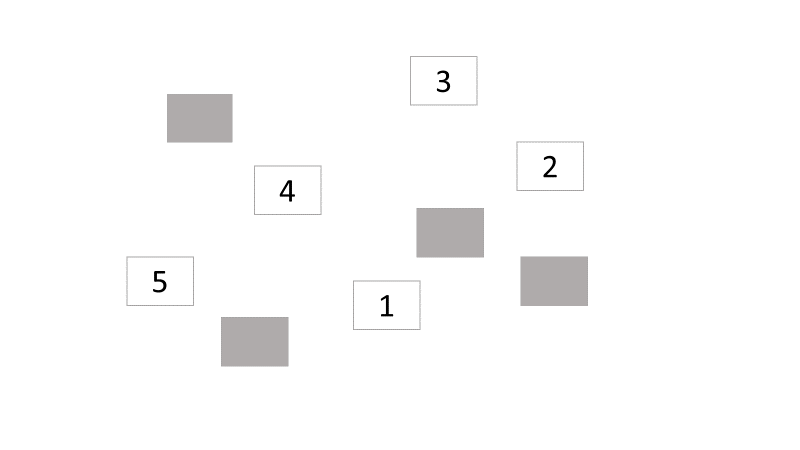


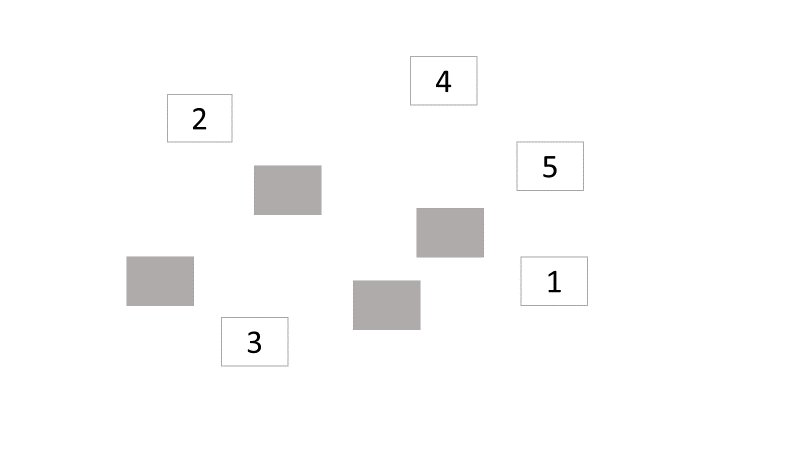


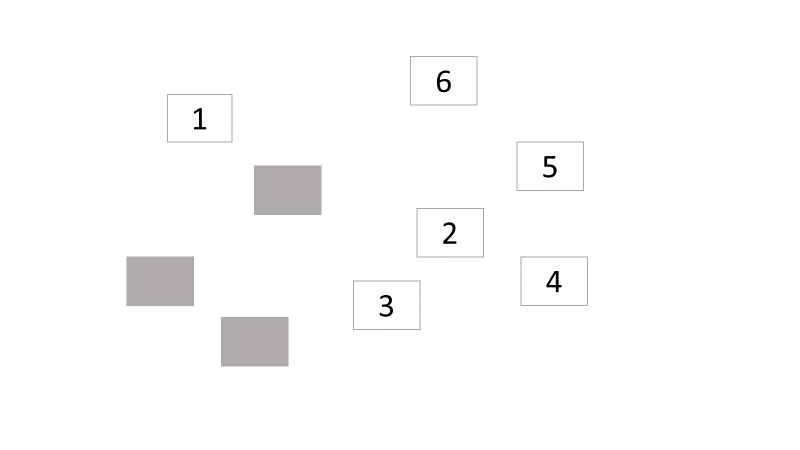


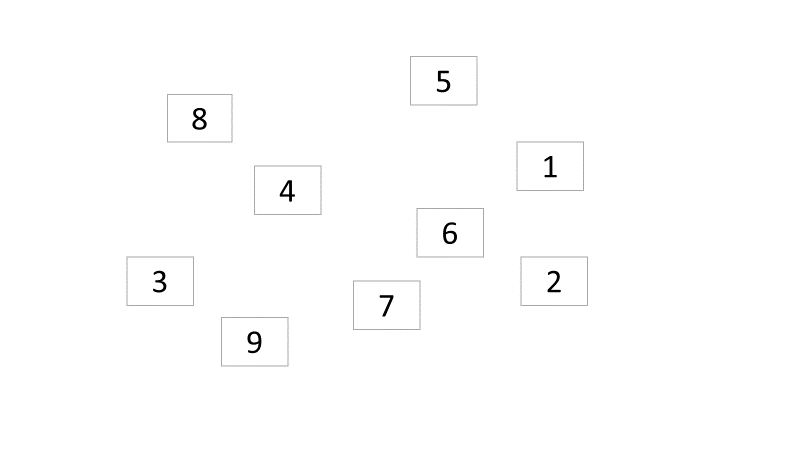

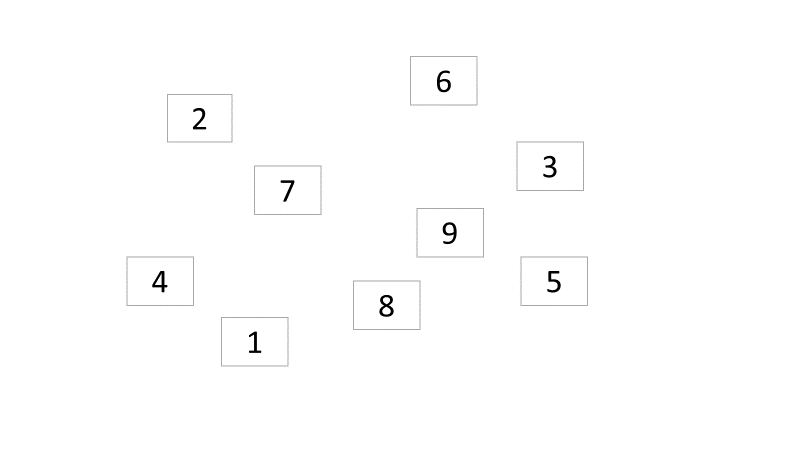

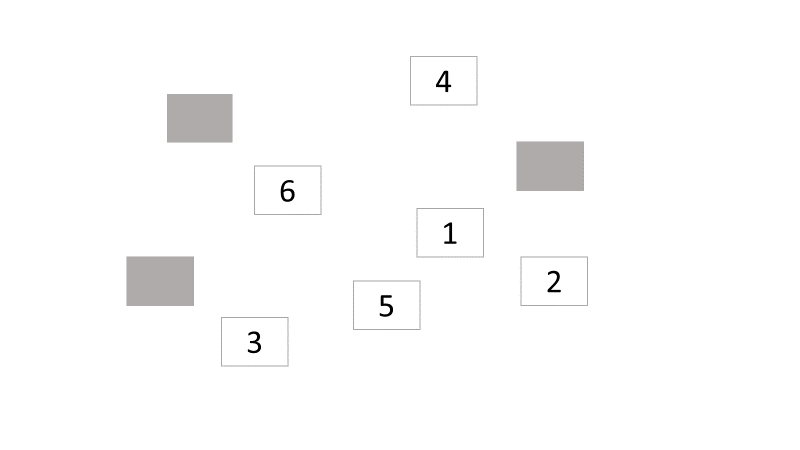

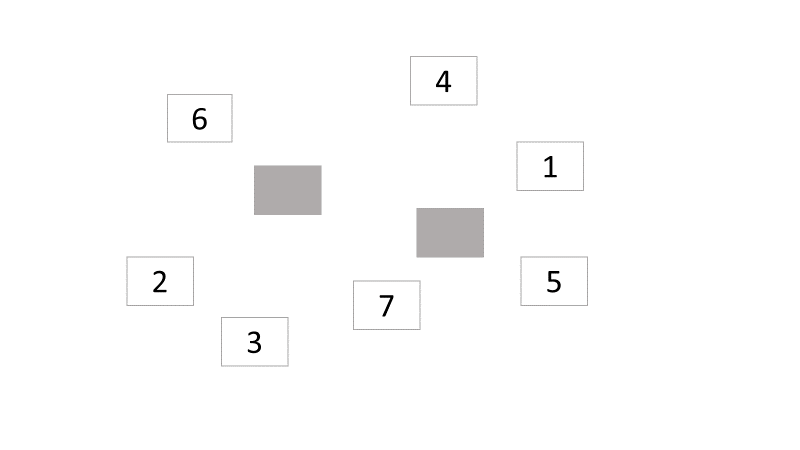

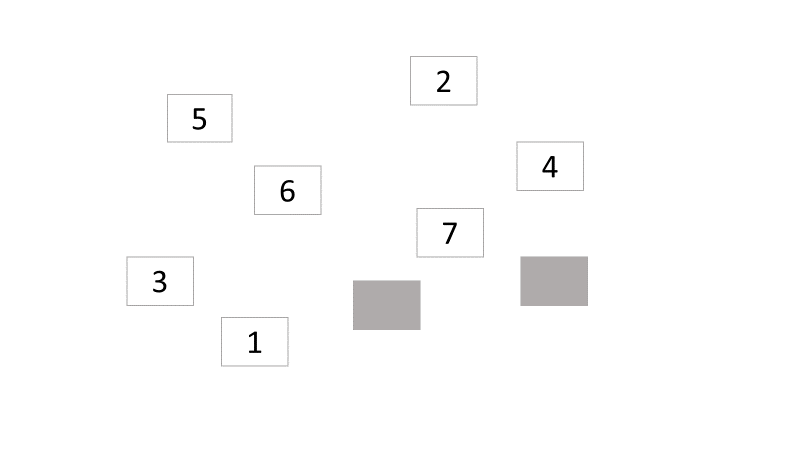

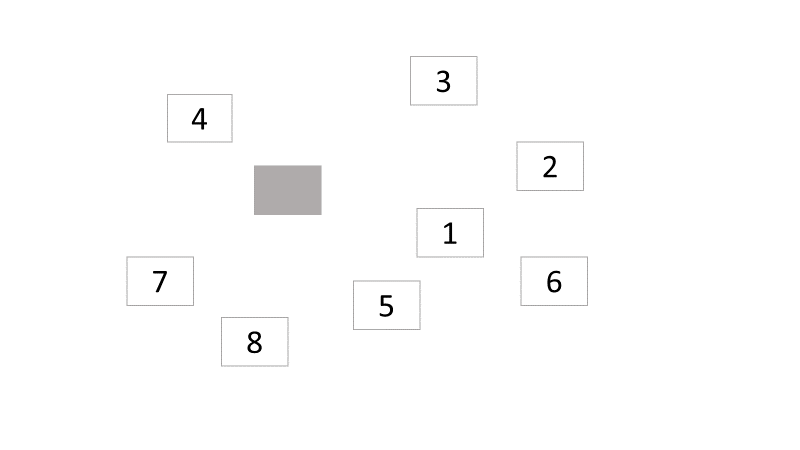

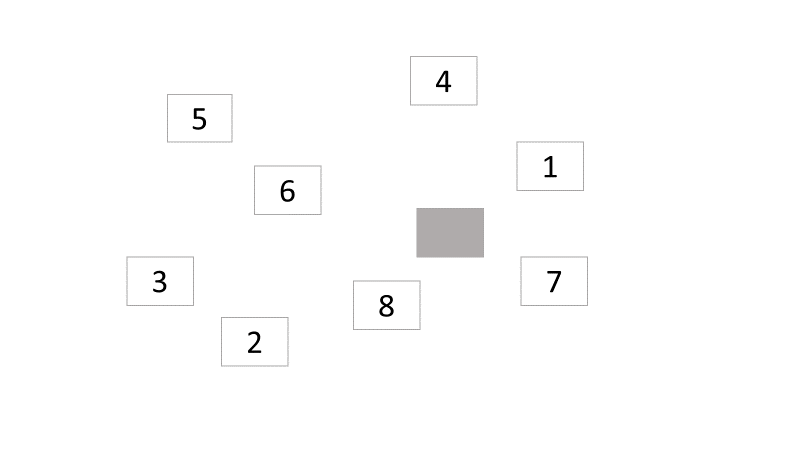

Supplement: Supplementary file 2 [file 10339_2024_1177_MOESM2_ESM.docx]
