# Supplementary material for: Chronic exercise and neuropsychological function in healthy young adults: a randomised controlled trial investigating a running intervention
Source: Cogn Process. 2024 Feb 29;25(2):241–58. doi: 10.1007/s10339-024-01177-1 (PMC11106121; doi:10.1007/s10339-024-01177-1)
Supplement: Supplementary file 3 [file 10339_2024_1177_MOESM3_ESM.docx]

**Online Resource 3**

**Record of Exercise (Week 1)**

**Please record your exercise sessions below. Exercise should elevate your heart and breathing rate. For those who have been asked to exercise should you miss a session it is very important to note this down! Please email back to me at the end of each week or if you have any questions.**

Date: Time:

Length (Time): Length (distance):

Please provide details of the location (try to be as detailed as possible): e.g. ran three times around the track next to Logan Park High school

Date: Time:

Length (Time): Length (distance):

Please provide details of the location (try to be as detailed as possible): e.g. ran three times around the track next to Logan Park High school

Date: Time:

Length (Time): Length (distance):

Please provide details of the location (try to be as detailed as possible): e.g. ran three times around the track next to Logan Park High school

**If you do any additional exercise, please record that below. Please copy and paste the details from above and fill in using the same format.**
