# Supplementary material for: Chronic exercise and neuropsychological function in healthy young adults: a randomised controlled trial investigating a running intervention
Source: Cogn Process. 2024 Feb 29;25(2):241–58. doi: 10.1007/s10339-024-01177-1 (PMC11106121; doi:10.1007/s10339-024-01177-1)

**Online Resource 4**

**Table S1.**

*Change scores for indoor (n = 15) versus outdoor (n = 6) runners and statistics for environment RM ANOVA interactions.*

| **MEASURE** | **INDOOR CHANGE SCORE** | **OUTDOOR CHANGE SCORE** | **F-STATISTIC** | **P-VALUE** | **EFFECT SIZE** |
| --- | --- | --- | --- | --- | --- |
| Pro | 4.29 (49.5) | -6.67 (30.91) | 0.30 | .592 | 0.02 |
| Anti | -0.67 (35.68) | -3.33 (27.49) | 0.05 | .821 | 0.00 |
| Pro/Anti | -36.00 (52.38) | -46.67 (63.16) | 0.22 | .645 | 0.01 |
| Forward Spatial | 4.20 (19.95) | 19.00 (18.81) | 3.54 | .075 | **0.16** |
| Backward Spatial | 5.67 (31.28) | 1.83 (8.73) | 0.04 | .833 | 0.00 |
| Forward Digit | 6.00 (28.03) | 2.17 (30.05) | 0.07 | .795 | 0.00 |
| Backward Digit | 3.73 (15.98) | 3.17 (10.98) | 0.01 | .940 | 0.00 |
| Positive Affect | -0.27 (4.78) | 2.33 (5.41) | 1.06 | .316 | 0.05 |
| Negative Affect | -0.13 (3.67) | -4.00 (4.08) | 4.04 | .059 | **0.18** |
| Fitness | -0.03 (3.22) | -0.46 (1.65) | 0.09 | .774 | 0.00 |
| Running Enjoyment^†^ | 61.00 (18.57) | 66.67 (12.32) | 0.15 | .520 | — |

*Note*. ^†^Enjoyment scores are means at Time 2; Pearson’s correlation analysed running enjoyment, thus the test statistic is *r*, which serves as the effect size. At least medium effect sizes (*ƞp^2^* ≥ 0.06) are **bolded**.

**Table S2***.*

*Pearson’s r correlation coefficients and p-values for relationship between running enjoyment and fitness, cognitive and affect change scores and running adherence (n = 24).*

| **MEASURE** | **PEARSON CORRELATION COEFFICIENT** | **P-VALUE** |
| --- | --- | --- |
| Pro | .088 | .682 |
| Anti | -.053 | .805 |
| Pro/Anti | -.163 | .446 |
| Forward Spatial | .076 | .723 |
| Backward Spatial | -.029 | .895 |
| Forward Digit | **-.580** | .003 |
| Backward Digit^†^ | -.082 | .709 |
| Positive Affect | **.339** | .105 |
| Negative Affect | -.004 | .987 |
| Fitness | .188 | .987 |
| Running Adherence | .181 | .409 |

*Note*. At least medium effect sizes (*r* ≥ 0.3) are **bolded**. ^†^*n* = 23

**Figure S1**

*Scatterplot illustrating the relationship between running enjoyment and Forward Digit change score (n = 24).*


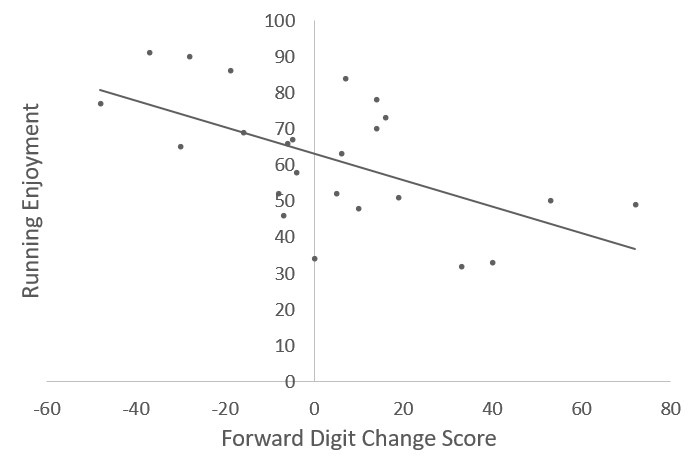

Supplement: Supplementary file 4 [file 10339_2024_1177_MOESM4_ESM.docx]
